# Mitogenomic sequences and evidence from unique gene rearrangements corroborate evolutionary relationships of myctophiformes (Neoteleostei)

**DOI:** 10.1186/1471-2148-13-111

**Published:** 2013-06-03

**Authors:** Jan Y Poulsen, Ingvar Byrkjedal, Endre Willassen, David Rees, Hirohiko Takeshima, Takashi P Satoh, Gento Shinohara, Mutsumi Nishida, Masaki Miya

**Affiliations:** 1 Natural History Collections, University Museum of Bergen, University of Bergen, Allégaten 41, P.O. Box 7800, Bergen N-5020, Norway; 2 Department of Marine Bioscience, Atmosphere and Ocean Research Institute, The University of Tokyo, 5-1-5 Kashiwanoha, Kashiwa-shi, Chiba 277-8564, Japan; 3 National Museum of Nature and Science, 4-1-1 Amakubo, Tsukuba, Ibaraki 305-0005, Japan; 4 Department of Zoology, Natural History Museum and Institute, 955-2 Aoba-cho, Chuo-ku, Chiba 260-8682, Japan

**Keywords:** Myctophiformes, Myctophidae, Neoscopelidae, Phylogeny, Mitogenomics, Gene rearrangements, Non-coding sequence

## Abstract

**Background:**

A skewed assemblage of two epi-, meso- and bathypelagic fish families makes up the order Myctophiformes – the blackchins Neoscopelidae and the lanternfishes Myctophidae. The six rare neoscopelids show few morphological specializations whereas the divergent myctophids have evolved into about 250 species, of which many show massive abundances and wide distributions. In fact, Myctophidae is by far the most abundant fish family in the world, with plausible estimates of more than half of the oceans combined fish biomass. Myctophids possess a unique communication system of species-specific photophore patterns and traditional intrafamilial classification has been established to reflect arrangements of photophores. Myctophids present the most diverse array of larval body forms found in fishes although this attribute has both corroborated and confounded phylogenetic hypotheses based on adult morphology. No molecular phylogeny is available for Myctophiformes, despite their importance within all ocean trophic cycles, open-ocean speciation and as an important part of neoteleost divergence. This study attempts to resolve major myctophiform phylogenies from both mitogenomic sequences and corroborating evidence in the form of unique mitochondrial gene order rearrangements.

**Results:**

Mitogenomic evidence from DNA sequences and unique gene orders are highly congruent concerning phylogenetic resolution on several myctophiform classification levels, corroborating evidence from osteology, larval ontogeny and photophore patterns, although the lack of larval morphological characters within the subfamily Lampanyctinae stands out. Neoscopelidae is resolved as the sister family to myctophids with *Solivomer arenidens* positioned as a sister taxon to the remaining neoscopelids. The enigmatic *Notolychnus valdiviae* is placed as a sister taxon to all other myctophids and exhibits an unusual second copy of the tRNA-Met gene – a gene order rearrangement reminiscent of that found in the tribe Diaphini although our analyses show it to be independently derived. Most tribes are resolved in accordance with adult morphology although Gonichthyini is found within a subclade of the tribe Myctophini consisting of ctenoid scaled species. Mitogenomic sequence data from this study recognize 10 reciprocally monophyletic lineages within Myctophidae, with five of these clades delimited from additional rearranged gene orders or intergenic non-coding sequences.

**Conclusions:**

Mitogenomic results from DNA sequences and unique gene orders corroborate morphology in phylogeny reconstruction and provide a likely scenario for the phylogenetic history of Myctophiformes. The extent of gene order rearrangements found within the mitochondrial genomes of myctophids is unique for phylogenetic purposes.

## Background

Lanternfishes (Myctophidae) and blackchins (Neoscopelidae) comprise the only two families in the order Myctophiformes, superorder Scopelomorpha [1], a group currently including some 250 species [2]. The 32 genera of Myctophidae contain about 98% of the species diversity, and morphological characters have clearly shown Myctophidae more derived compared to the more generalized Neoscopelidae [3-6]. Myctophids are exclusively marine and pelagic, occupying depths from surface waters down to the upper 1000 meters of the bathypelagic layer (1000–4000 meters). The group as a whole constitutes fairly small fishes ranging in size from 20 to 300 millimeters, with larger members being confined to the bathypelagic realm. They are ubiquitous in the World’s oceans and exhibit extremely high abundances with one estimate as high as 65% of all pelagic deep-sea fish biomass [7].

**Figure 1:**
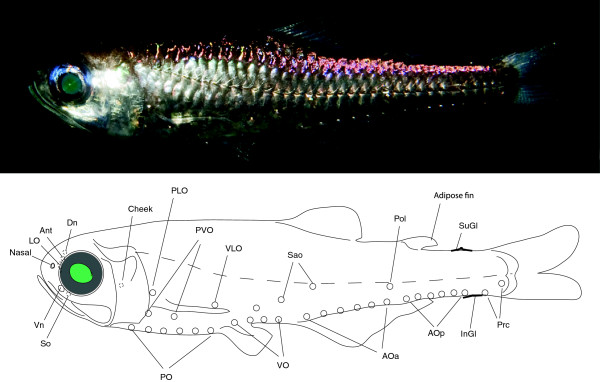
***Benthosema glaciale, *© Rudolf Svendsen, Høgsfjorden, Norway.** Standard myctophid photophore terminology noted with photophores/luminescent organs truncated if found in other myctophids than *B. glaciale*. Abbreviations: Ant – Anterior orbital (antorbital), AOa – Anal organs anterior, AOp – Anal organs posterior, Dn – Dorsal nasal (dorsonasal), InGl – Infracaudal gland, LO – Luminous organ, PO – Pectoral organs, Pol – Posterior organ lateral, PLO – Pectoral lateral organ, Prc – Precaudal organs (precaudals), PVO – Pectoral ventral organs, Sao – Supraanal organs, So – Suborbital, Gl – Supracaudal gland, VLO – Ventral lateral organ, Vn – Ventral nasal (ventronasal), VO – Ventral organs.

The most striking feature of myctophids is their often species-specific patterns of photophores (light-organs), which have earned them their popular name lanternfishes. Photophores are named according to specific patterns present in various degrees throughout different myctophid lineages (Figure 1), making photophore patterns important when reconstructing the evolution of myctophids. It is generally believed that species-specific flash communication underpins the evolution of photophore patterns although myctophids are also macrosmatic, i.e. possessing large olfactory organs, indicating a complicated communication system not fully understood [8]. Myctophid fishes are believed to communicate in a firefly-like manner by using duration- and intensity-variable flashes of light produced by species-specific and sexually dimorphic patterned photophores [3,9]. One argument against flash communication is the almost identical photophore patterns observed between some species, making it virtually impossible to discriminate between flashes except within very close range [10]. Although bioluminescence has evolved multiple times across open-ocean organisms [11], the possibility of using this feature in tracing the natural history of an entire clade is, however, unique within open-ocean and deep-sea animals. The mechanism behind myctophid bioluminescence has caused some debate since bioluminescent bacteria of the strain *Vibrio* were found by Foran [12] using hybridization probes and later challenged by Haygood et al. [13] who confidently excluded both symbiotic bacteria and bacterial luciferase as the light source. It is now generally accepted that a coelenterazine system is responsible for myctophid bioluminescence [11,14], contrary to e.g. the famous deep-sea anglerfishes that possess family-specific symbiotic photobacterial strains of *Vibrio* in the esca [15,16]. Most myctophids that have been examined concerning their visual spectrum show a tendency towards longer wavelengths for absorption and emission, resulting in a dim blue bioluminescence as typically found in deep-sea fishes [17]. The latter study found a relatively confined spectral range of absorption in myctophids compared to other mesopelagic fishes. However, Hasegawa et al. [18] found that *Myctophum nitidulum* also possessed longer wavelength retinal pigments enabling them to detect a broader spectrum of wavelengths produced by other animals, and/or their own light emission and down-welling sunlight. Extended spectral range is familiar from loose-jaw dragonfishes well-known for emitting long-wavelength red light from orbital photophores, enabling them to communicate or detect prey at wavelengths invisible to other deep-sea organisms [19]. Turner et al. [17] note that possession of a relatively limited spectral range could be the result of a shared evolutionary history more than multiple instances of visual adaptation to the mesopelagic environment.

Diel vertical migration (DVM) is a prominent feature of myctophids although not all myctophid species engage in this activity [20]. DVM prompted the famous “false bottom” (or the deep scattering layer) discovery, turning out to be massive occurrences of the swimbladder-possessing myctophid *Ceratoscopelus maderensis* among other deep-sea animals [21]. DVM is common across many animal groups and is generally believed to involve feeding and/or reduced predation risk associated with reduced light intensity [22]. Herring [23] notes that if populations are biased towards horizontal layers, as is often the case with myctophids, DVM might be an effective way to channel intra-specific encounters and could be important in mate recognition. A phylogenetic component in DVM has not been explored although DVM is clearly more pronounced in some groups of myctophids than others, e.g. the slendertails of the tribe Gonichthyini including the genera *Gonichthys, Centrobranchus, Tarletonbeania* and *Loweina*.

**Figure 2:**
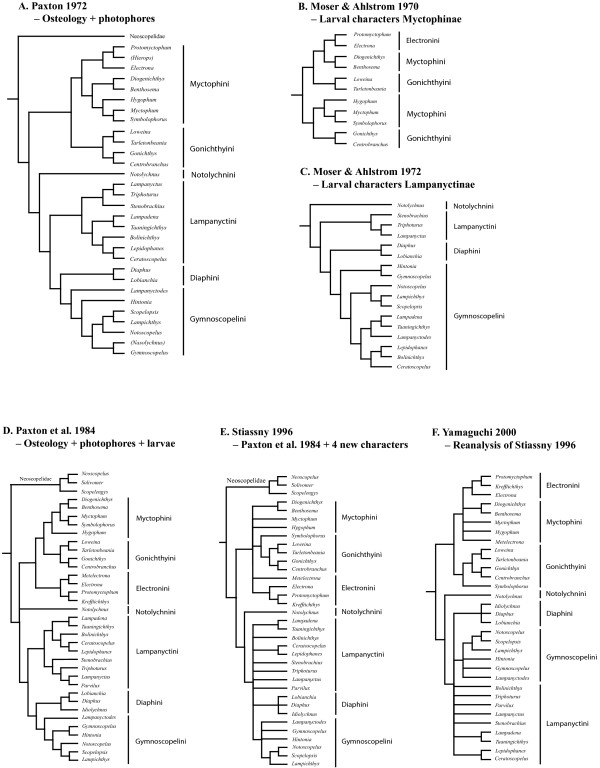
**Phylogenetic hypotheses from previous morphological studies. A**) Adult osteology [3]. Parenthesized genera no longer recognized. **B**) Larval development (Myctophinae only) with topology constructed from written considerations by Moser & Ahlstrom [27] and tribes inferred according to [3]. **C**) Larval development (Lampanyctinae only) [28]. **D**) Adult osteology + larval development [30]. **E**) Unordered multistate parsimony analysis of adult + larval characters including 4 new characters [5]. **F**) Ordered multistate parsimony reanalysis of all adult and larval characters [24].

Stiassny [5] and Yamaguchi [24] have most recently reviewed myctophiform synapomorphies and there is little doubt that the order constitutes a monophyletic assemblage. Intrarelationships of Neoscopelidae (3 genera and 6 species) are not well resolved [5] with generic relationships within Myctophidae better understood bearing on the classification initially by Bolin [6], subsequently Fraser-Brunner [4], and finally Paxton [3]. They classified the order into two families, divided the myctophids into two subfamilies that together include six tribes, based on osteology and photophore patterns (Figure 2A). This classification is still in use today, except for the lineage Electronini also being recognized as a distinct tribe as originally designated by Wisner [25]. Additional corroborating evidence for this classification has been presented partly from urodermal bones [26] and notably a broad range of larval body forms [27-30] (Figure 2B-D). Myctophid larvae show some of the most diverse forms within any teleost order and characters associated with early ontogeny have proven valuable in phylogenetics [30]. Stiassny [5] added new data and reviewed existing characters, resulting in a topology little resolved below subfamily level (Figure 2E). Yamaguchi [24] carried out the most recent analysis using the characters provided by the previous studies, which again resulted in an unresolved topology (Figure 2F). Morphological work on photophore patterns, adult osteology and larval ontogeny have clearly established their utility in myctophid phylogenetics, although missing larval characters in the subfamily Lampanyctinae have been a significant source of discrepancy between morphological hypotheses [29]. Larval characters corroborate parts of adult phylogenetic reconstruction (Figure 2) although the larvae of the large genus *Diaphus* have proven exceptionally difficult to identify [30]. The most persistent issue concerning myctophid phylogenetics has been the placement of *Notolychnus valdiviae*, a monotypic, diminuitive, putatively plesiomorphic myctophid showing dorsally located photophores, weak ossification and several characters difficult to assess even on subfamily level [3]. Recent cladistic analyses of morphological characters have converged on *Notolychnus* as sister taxon to the remaining lampanyctines (Figure 2) as initially suggested by Paxton [3].

Surprisingly little molecular work has been carried out on Myctophiformes considering their abundance and at times problematic species identification. Yamaguchi et al. [31] tested photophore patterns, biogeographical distributions and diverse larval eye morphology within *Hygophum* in a phylogenetic context. The results indicated all three attributes as somewhat informative concerning speciation pattern, although clear conclusions were hard to extract due to few characters (partial 16S rRNA gene sequences) and incomplete taxon sampling. However, with an increasing understanding of myctophiform phylogeny, this type of study will become highly valuable in terms of assessing open-ocean speciation patterns in myctophids as well as other open-ocean fish components [32].

Miya et al. [33] produced the first mitochondrial (mt) genome mapping of myctophiform fishes, showing that the two myctophids *Diaphus splendidus* and *Myctophum affine* possess non-typical vertebrate mt gene arrangements, whereas the neoscopelid fish *Neoscopelus microchir* exhibits the typical gene arrangement for vertebrates [34]. Their findings indicated that mt gene arrangements of myctophid fishes have the potential to provide evidence concerning clade delimitation at various levels. Clade-specific gene orders are considered strong evidence for monophyly since the process of gene duplication and subsequent deletions are considered random and rare events [35-39]. However, accumulating evidence shows positive selection on some rearranged mt gene orders [40] to be much more common than initially expected [41]. Intergenic non-coding (INC) regions are likewise believed to provide evidence for phylogenetic history, although this group of markers has been poorly explored mainly because of insufficient taxon sampling.

This study is the first to use mitogenomic DNA sequences combined with additional information from extensively rearranged gene orders and INC-sequences to explore myctophiform phylogeny.

## Methods

### Taxon sampling

Twenty-three genera and a total of 41 species of myctophiform fishes are included in this study – 38 species representing new data and three from a previous mitogenomic study on higher teleostean phylogeny [33]. Forty-six outgroup taxa ranging from early euteleost groups (root) to ophidiiform and lophiiform fishes (Percomorpha) were chosen with all putative myctophiform sister groups included. Taxonomic sampling focused on tribal classification sensu Paxton [3] representing the distinct myctophiform lineages. We sampled all non-monotypic tribes with multiple genera although only a single representative of Gonichthyini is included due to technical difficulties in amplification of DNA. A large number of taxa within the genus *Diaphus* were included since about 1/3 of all myctophid species are placed within this genus. Voucher specimens were obtained from sampling onboard Research Vessel Pâmiut (Greenland Institute of Natural Resources) around Greenland (JYP), from the MAR-ECO cruise (IB), catalogued and uncatalogued material at NSMT (TPS and GS) and institutional loans from ASIZP, BSKU, KU, MCZ, SIO, WU and ZMUC (Table 1). Voucher specimens were examined and photographed with e-vouchers available online [42].

**Table 1:** 87 taxa included in this study

**Clade**	**Species**	**Acc. #**	**Tissue #**	**Voucher Museum #**	**Study**
**Esociformes**	***Esox lucius***	AP004103			Ishiguro et al. 2003 [43]
	***Dallia pectoralis***	AP004102			Ishiguro et al. 2003 [43]
**Salmoniformes**	***Coregonus lavaretus***	AB034824			Miya & Nishida 2000 [44]
	***Thymallus thymallus***	FJ853655			Yasuike et al. 2010
	***Salvelinus alpinus***	AF154851			Doiron et al. 2002
**Argentiniformes**	***Bathylagus ochotensis***	AP004101			Ishiguro et al. 2003 [43]
	***Nansenia ardesiaca***	AP004106			Ishiguro et al. 2003 [43]
	***Glossanodon semifasciatus***	AP004105			Ishiguro et al. 2003 [43]
	***Opistophroctus soleatus***	AP004110			Ishiguro et al. 2003 [43]
**Osmeriformes**	***Retropinna retropinna***	AP004108			Ishiguro et al. 2003 [43]
	***Salangichthys microdon***	AP004109			Ishiguro et al. 2003 [43]
	***Mallotus villosus***	HM106491			Li et al. 2010
	***Plecoglossus altivelis***	AB047553			Ishiguro et al. 2001
**Stomiiformes**	***Diplophos taenia***	AB034825			Miya & Nishida 2000 [44]
	***Chauliodus sloani***	AP002915			Miya et al. 2001 [33]
	***Gonostoma gracile***	AB016274			Miya & Nishida 1999 [45]
**Galaxiiformes**	***Galaxiella nigrostriata***	AP006853			Miya et al. (unpubl.)
	***Galaxias maculates***	AP004104			Ishiguro et al. 2003 [43]
	***Galaxias gollumoides***	HM106487			Li et al. 2010
**Aulopiformes**	***Synodus variegatus***	AY524977			Chen & Wu (unpubl.)
	***Chlorophthalmus agassizi***	AP002918			Miya et al. 2001 [33]
	***Aulopus japonicus***	AB047821			Kawaguchi et al. 2001 [33]
	***Harpadon microchir***	AP002919			Miya et al. 2001 [33]
	***Saurida undosquamis***	AP002920			Miya et al. 2001 [33]
**Ateleopodiformes**	***Ateleopus japonicus***	AP002916			Miya et al. 2001 [33]
	***Ijimaia dofleini***	AP002917			Miya et al. 2001 [33]
**Lampriformes**	***Lampris guttatus***	AP002924			Miya et al. 2001 [33]
	***Zu cristatus***	AP002926			Miya et al. 2001 [33]
	***Trachipterus trachypterus***	AP002925			Miya et al. 2001 [33]
**Myctophiformes**	***Neoscopelus macrolepidotus***	AP012238	KU 3297	Uncatalogued	This study
	***Neoscopelus microchir***	AP002921			Miya et al. 2001 [33]
	***Scopelengys tristis***	AP012228	NSMT-P 99997	NSMT-P 99997	This study
	***Solivomer arenidens***	AP012249	NSMT	Uncatalogued	This study
	***Benthosema fibulatum***	AP012253	NSMT-P 75816	NSMT-P 75816	This study
	***Benthosema glaciale***	AP012264	ZMUC #8160	ZMUC P2393965	This study
	***Benthosema pterotum***	AP012260	NSMT-P 75855	NSMT-P 75855	This study
	***Bolinichthys distofax***	AP012232	NSMT-P 97624	NSMT-P 97624	This study
	***Bolinichthys pyrsobolus***	AP012261	NSMT-P 92273	NSMT-P 92273	This study
	***Centrobranchus choerocephalus***	AP012237	NSMT-P 102902	NSMT-P 102902	This study
	***Ceratoscopelus maderensis***	AP012259	ZMUC #6600	ZMUC P2393929	This study
	***Diaphus chrysorhynchus***	AP012230	NSMT-P 79902	NSMT-P 79902	This study
	***Diaphus gigas***	AP012235	NSMT-P 91530	NSMT-P 91530	This study
	***Diaphus luetkeni***	AP012231	ASIZP 911514	ASIZP66276	This study
	***Diaphus splendidus***	AP002923			Miya et al. 2001 [33]
	***Diaphus theta***	AP012240	KU 2135	KU 27971	This study
	***Diogenichthys atlanticus***	AP012233	SIO 09320	SIO 09320	This study
	***Electrona antarctica***	AP012248	ZMUC #7552	ZMUC P2393962	This study
	***Gymnoscopelus nicholsi***	AP012250	ZMUC #7806	ZMUC P2393963	This study
	***Krefftichthys anderssoni***	AP012236	ZMUC #7550	ZMUC P2393964	This study
	***Lampadena anomala***	AP012227	ME 4201#3	ZMUB 18019	This study
	***Lampadena urophaos atlantica***	AP012251	ME 7371#4	ZMUB 18050	This study
	***Lampadena yaquinae***	AP012257	NSMT-P 72341	NSMT-P 72341	This study
	***Lampanyctus macdonaldi***	AP012241	ZMUC #8077	ZMUC P2393967	This study
	***Lampanyctus crocodilus***	AP012258	ZMUC #8076	ZMUC P2393968	This study
	***Lobianchia gemellarii***	AP012242	ME 4239#6	ZMUB O.1725	This study
	***Myctophum affine***	AP002922			Miya et al. 2001 [33]
	***Myctophum asperum***	AP012234	NSMT-P 91490	NSMT-P 91490	This study
	***Myctophum nitidulum***	AP012255	NSMT-P 92345	NSMT-P 92345	This study
	***Myctophum orientale***	AP012254	NSMT-P 77350	NSMT-P 77350	This study
	***Myctophum punctatum***	AP012239	ZMUC #6594	ZMUC P2393969	This study
	***Nannobrachium ritteri***	AP012247	KU 2237	KU 28276	This study
	***Notoscopelus caudispinosus***	AP012256	KU 5301	MCZ 161883	This study
	***Notoscopelus japonicus***	AP012252	BSKU 103772	BSKU 103772	This study
	***Notoscopelus kroyeri***	AP012262	ZMUC #8078	ZMUC P2393970	This study
	***Notolychnus valdiviae***	AP012229	NSMT-P 102930	NSMT-P 102930	This study
	***Protomyctophum arcticum***	AB648909-10	ZMUC #8476	ZMUC P2393971	This study
	***Stenobrachius leucopsarus***	AP012245	NSMT-P 78748	NSMT-P 78748	This study
	***Symbolophorus californiensis***	AP012246	NSMT-P 92257	NSMT-P 92257	This study
	***Taaningichthys minimus***	AP012244	ME 5633	ZMUB 18049	This study
	***Triphoturus nigrescens***	AP012243	NSMT-P 102934	NSMT-P 102934	This study
**Ophidiiformes**	***Cataetyx rubrirostris***	AP004407			Miya et al. 2003 [46]
	***Carapus bermudensis***	AP004404			Miya et al. 2003 [46]
	***Bassozetus zenkevitchi***	AP004405			Miya et al. 2003 [46]
**Lophiiformes**	***Sladenia gardineri***	AB282827			Miya et al. 2010 [47]
	***Coelophrys brevicaudata***	AB282834			Miya et al. 2010 [47]
	***Thaumatichthys pagidostomus***	AB282847			Miya et al. 2010 [47]
**Percopsiformes**	***Percopsis transmontana***	AP002928			Miya et al. 2001 [33]
	***Aphredoderus sayanus***	AP004403			Miya et al. 2003 [46]
**Polymixiiformes**	***Polymixia japonica***	AB034826			Miya & Nishida 2000 [44]
	***Polymixia lowei***	AP002927			Miya et al. 2001 [33]
**Gadiformes**	***Stylephorus chordatus***	AB280688			Miya et al. 2007
	***Bregmaceros transmontana***	AP004411			Miya et al. 2003 [46]
	***Trachyrincus murrayi***	AP008990			Satoh et al. 2006 [48]
	***Lota lota***	AP004412			Miya et al. 2003 [46]
**Zeiformes**	***Zeus faber***	AP002941			Miya et al. 2001 [33]
	***Parazen pacificus***	AP004433			Miya et al. 2003 [46]
	***Zenion japonicum***	AP004434			Miya et al. 2003 [46]

Taxa included in this study listed according to ordinal classification. Mitogenome accession number, tissue number, specimen + institute number and study noted if information available.

### Production of mitogenomic data

Genomic DNA was extracted using the Qiagen Puregene extraction kit following the manufacturer´s protocol and used directly for long and accurate amplification PCR (LA PCR) of the entire mitochondrial genome [49]. Depending on tissue quality and LA primer fidelity, a variable number of LA PCR reactions were needed for complete coverage, with a minimum of two and a maximum of six for some species. The LA PCR product was diluted with ddH_2_O according to success of amplification and nested short PCR was subsequently performed on the LA product with a broad range of fish universal and species specific primers (>200). Double stranded PCR products were cleaned with Exo-Sap at 60°C for 60 minutes and used as template for direct cycle-sequencing with dye labeled terminators (Applied Biosystems). All fragments were sequenced on automated DNA sequencers according to length of labelled fragments. Quality of tissue was shown to be a prominent obstacle regarding successful amplifications. Issues regarding amplification necessitated multiple PCR protocols and tailor-made primer sets for different taxa. Variable number tandem repeats found in the control region and various INC-regions added to the technical issues. Primer information and protocols regarding PCR and sequencing for particular species are available by request (JYP). Newly determined mitogenome sequences are available as [DDBJ, EMBL or GenBank AP012227-62, AP0122264 and AB648909-10] (Table 1).

### Sequence editing, alignment and analyses

Raw sequence data were processed and formatted with the software 4peaks Ver. 1.7.2 [50], Textwrangler Ver. 3.5.3 [51] and MacClade Ver. 4.06 [52] at various stages before alignment. Mitogenome assembly was performed using Sequencher Ver. 4.10.1 [53] by concatenation of overlapping sequences. Numerous single nucleotide repeat regions were concatenated from the L- and H-strand sequences assuming the repeat region to be identical in the two strands. This was verified by electrophoresis of the sequenced PCR products showing bands with equal size to the sequenced fragments. All genes were initially annotated by alignment to those from closely related fishes already available in GenBank [54]. Subsequently, protein coding genes were examined by reference to ORFs and the 22 tRNA genes were examined for secondary structure by tRNAscan-SE Ver. 1.21 [55,56]. The two ribosomal RNA genes (12S and 16S) were annotated as the sequences between flanking tRNA Phe, Val and Leu^UUR^ in addition to alignment using previously determined myctophid RNA genes [33].

Alignment of the 12 protein coding genes was done by eye whereas the 22 tRNA genes and 12S+16S rRNA genes were aligned with a probabilistic multiple alignment approach using ProAlign Ver. 0.5 [57]. The ND6 gene was excluded due to heterogeneous base composition [44] leaving 10908 protein coding nucleotide characters (3636 amino acids). Ambiguous alignment of the tRNA and rRNA genes were discarded from the analyses based on the 90% minimum posterior probability of correct alignment implemented in ProAlign, leaving 938 and 1474 nucleotide characters from 22 tRNA and 12S+16S rRNA genes, respectively. All genes were aligned separately and subsequently concatened into one dataset consisting of six partitions as suggested by PartitionFinder [58] (see Additional file 1); amino acids, 12S+16S rRNA genes and 22 concatenated tRNA genes (designated as 123_A_RT_n_). Missing nucleotides, e.g. tRNA-Thr and Pro genes for some species, were coded as missing characters. Levels of mutational saturation were calculated from p-distances for 1st, 2nd and 3rd codon positions using PAUP* Ver. 4.0b10 [59] (see Additional file 2). Since no mutational saturation was detected when including only myctophiforms, we constructed a second dataset consisting of only nucleotides from the 41 myctophiform species. This dataset included all mitogenomic nucleotides except the CR and ProAlign ambiguous alignment as already noted above (123_n_RT_n_).

Heuristic maximum likelihood phylogenetic analyses with 1000 non-parametric bootstrap replications for all nodes were performed in a single run with the sequential version of RAxML Ver. 7.2.8 [60,61] under the GTR+Γ+I substitution model [62] as chosen by Akaike and Bayesian information criteria [63,64] implemented in Modelgenerator [65]. A final ML optimization of every 5th bootstrapped tree for the highest scoring ML-tree is performed by RAxML under the specified GTR+Γ+I substitution model [61]. Partitions constructed from amino acids were analyzed with the mtREV model [66] chosen by Modelgenerator.

Bayesian analysis was performed on the datasets with MrBayes Ver. 3.2.1 [67] using the same partitions and models as described above. Maximum likelihood analyses were conducted on a Quad-core Mac Pro desktop computer whereas the Bayesian analyses were computed on the Bioportal computer cluster University of Oslo [68]. Convergence of chains and burn-in were determined using TRACER Ver. 1.5 [69].

### Additional analyses and diaphini tRNA-met pseudogenes

All 41 myctophiform mitogenomes were scrutinized for mt features such as rearranged gene orders and INC-regions. All INC-sequences were analyzed for sequence similarity in relation to possible duplicated regions in the mitochondrial genome by using NCBI Blast [54], tRNAscan [55] and a series of decreasing assembly parameters in Sequencher [53]. Subsequently, alignments of INC-regions were examined for secondary folding patterns with LocARNA Ver. 1.5.2 [70] and RNAshapes Ver. 2.1.6 [71] in order to detect conserved regions. Secondary tRNA-structures were drawn with VARNA Ver. 3.8 [72]. In order to examine a putative associated gene order found in *Notolychnus* and species within the tribe Diaphini, neighbour-joining analyses were carried out including multiple myctophid tRNA-Met genes, putative duplicated diaphinid tRNA-Met genes (pseudogenes) and the two tRNA-Met genes observed in *Notolychnus*. Pairwise sequence variation of the pseudogenes and detected tRNA-Met genes in Diaphini were compared in order to validate the INC-regions as actual tRNA-Met pseudogenes, the latter expected to show higher substitution rates.

### Testing alternative phylogenetic hypotheses

Topology comparison from morphological phylogenies and this study were tested by using the approximately unbiased log-likelihood test [73] implemented in CONSEL [74]. Constrained trees were produced with Mesquite [75] and per-site log-likelihood scores were produced using RAxML using the f –g option with best scoring ML-trees passed via –z option. Per-site log-likelihood scores and tree topologies were used as direct input into CONSEL outputting tree-specific ML-scores and associated *p*-values.

## Results

### Mitogenome organization

**Figure 3:**
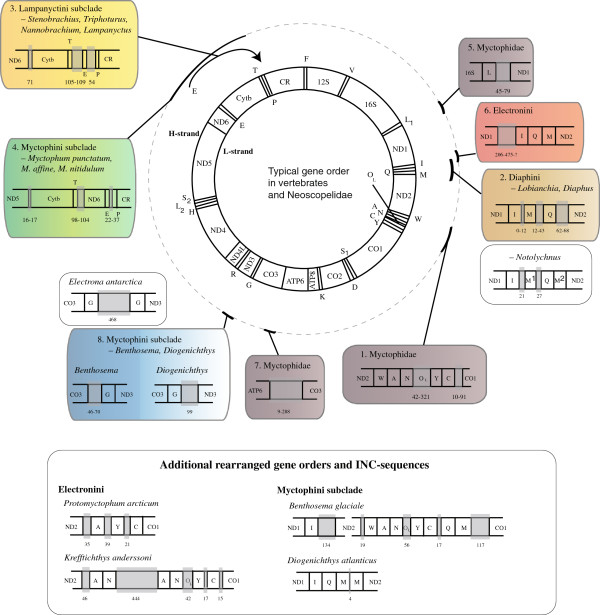
**Typical gene orders in vertebrates and Neoscopelidae with eight unique gene orders and INC-regions found in Myctophidae.** 1**–**4 show rearranged gene orders and 5–8 show INC-sequences with all eight features treated as clade-defining synapomorphies. Taxon-specific gene rearrangements are shown at the bottom. All INC-regions are shown in grey with number of base pairs noted if known. Mt information depicted is provided from all included taxa within the clades except for the rearrangement in 3 (information from *N. ritteri* and *L. crocodilus*), in 4 (information from *M. affine* and *M. nitidulum*) and in 6 (information from *K. anderssoni* and *E. antarctica*). The missing information for some included taxa is due to unsuccessful amplification of particular regions. All eight features are discussed in the text. Abbreviations follow common mt usage and colorcoding is signifying the same clades and defining gene orders throughout all figures in this article.

The organization of the mitogenome is unique in all fishes of the family Myctophidae, showing rearranged gene orders for either the whole family, tribal or subtribal clades, whereas Neoscopelidae show the canonical vertebrate gene order. All these features are summarized in Figure 3 with numbers 1–8 corresponding to rearranged gene orders (1–4) and INC-sequences (5–8).

1. WANYC-gene order found in all myctophids. The tRNA-Cys and Tyr have shifted positions and the putative O_L_-region was much longer than the usual vertebrate sequence in this region of about 30–40 base pairs (56–321 in myctophids included in this study). We were not able to find any conserved sequence blocks or secondary cloverleaf structure typical of tRNA genes [76] for this INC-region (see Additional file 3).

2. IMQM_ψ_ gene order found in the tribe Diaphini. The tRNA-Gln and Met have shifted positions with the typical L- and H-strand coding intact (tRNA-Gln is coded on the L-strand and tRNA-Met coded on the H-strand). An INC-region (tRNA-Met pseudogene denoted as M_ψ_) is present between tRNA-Gln and ND1 in all diaphinid taxa included. A similar gene order was also observed for *Notolychnus valdiviae* although the INC-region is a tRNA-Met gene for this taxon (IMQM gene order). This particular gene rearrangement was analyzed and is discussed in detail below.

3. Relocation of tRNA-Glu resulting in gene order Cytb/T/E/P in a Lampanyctini subclade. Species included within *Lampanyctus*, *Nannobrachium*, *Stenobrachius* and *Triphoturus* all show tRNA-Glu pseudogenes in the canonical position between ND6 and Cytb, verified from sequence comparisons and partial secondary cloverleaf structures (see Additional file 3). We were only able to determine the position of the tRNA-Glu gene in two of the five taxa, *Nannobrachium ritteri* and *Lampanyctus crocodilus,* all showing the novel position of tRNA-Glu between tRNA-Thr and Pro. A highly variable region downstream the CR and multiple nucleotide repeats prevented us from determining this sequence in *Lampanyctus macdonaldi*, *Stenobrachius leucopsarus* and *Triphoturus nigrescens*.

4. Cytb/T/ND6/E/P gene order in a Myctophini subclade. *Myctophum punctatum*, *M. affine* and *M. nitidulum* showed this rearranged gene order whereas *M. asperum* and *M. orientale* showed the canonical gene order. Unknown technical issues prevented us from determining the sequence of ND6 in *M. punctatum* although the gene rearrangement is evident also in this species from ND5/Cytb contiguous sequences as opposed to the canonical ND5/ND6/E/Cytb gene order.

5. INC-sequence between Leu^UUR^ and ND1 found in all myctophids. This spacer ranged from 45–79 base pairs and showed no secondary structure or sequence similarity to any other mt genes (see Additional file 3).

6. INC-sequence between ND1 and tRNA-Ile found in Electronini. This spacer showed significant length variation and we were unable to determine the whole sequence in *Protomyctophum arcticum*.

7. INC-sequence between ATP6 and CO3 found in all myctophids. This spacer was 288 base pairs long in *Notolychnus valdiviae* and ranged from 9–43 base pairs in all other myctophids. We found no mt gene similarity or secondary structure for this INC-region (see Additional file 3).

8. INC-sequence associated with tRNA-Gly found in a Myctophini subclade. This INC-region is treated as a character state for the clade comprising *Benthosema* and *Diogenichthys* although the exact position varies between the two genera. The three species of *Benthosema* included showed the INC-region located upstream the tRNA-Gly whereas in *Diogenichthys atlanticus*, the spacer was located downstream tRNA-Gly. *Electrona antarctica* showed a different organization with two detected tRNA-Gly genes separated by a large INC-region. We found no sequence similarity to other mt genes for this region or any secondary folding structures (see Additional file 3).

Additional gene orders and INC-sequences were observed for various myctophid species although none of the rearrangements were distributed across multiple taxa at this point. These additional gene order rearrangements and INC-sequences are also presented in Figure 3. Detailed information for rearranged gene orders such as length of INC-sequences and missing regions is presented in the supplementary material (see Additional file 4). The highly polymorphic control region (CR) was consistently problematic to amplify or sequence, resulting in parts of the CR missing for most species as well as flanking tRNA-Thr and -Pro for some species.

### Mitogenome sequences

**Figure 4:**
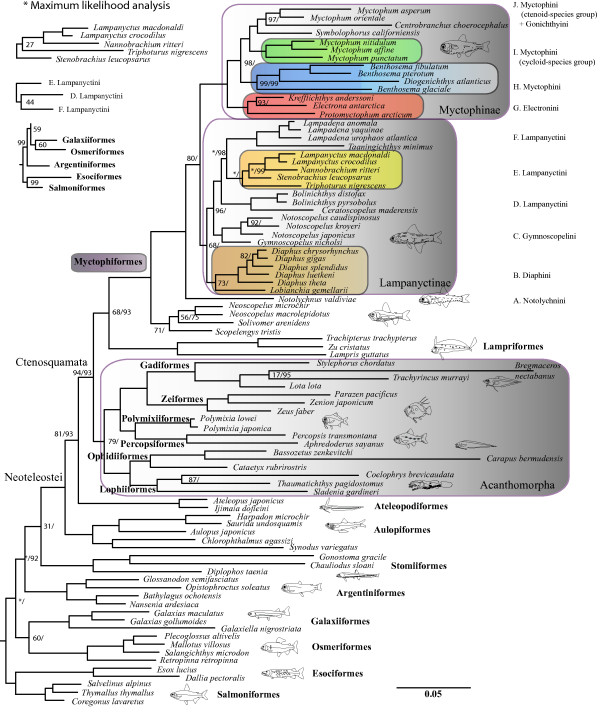
**Mitogenomic Neoteleostei phylogeny (123**_**A**_**RT**_**n**_**) rooted with Euteleostei.** Branch support from 1000 Bootstrap replicates and Bayesian posterior probabilities noted only if values are less than 100 – separated by a slash (**/**), respectively. Three topological differences between the Bayesian- and ML-analyses are noted with bootstrap values included. Ten distinct myctophid lineages are noted (A-J) with respect to current tribal classification.

ML- and Bayesian analyses of the mitogenomic dataset consisting of 87 taxa resolved a phylogenetic tree of eu- and neoteleostean orders as presented in Figure 4. Myctophiformes was resolved as the sister clade to Lampriformes supported by a BS-value of only 68% whereas the BPP-value was 93%. Both analyses conducted for this study showed this relationship; however, considering the long basal branches of Lampriformes and Myctophiformes, we conclude that the phylogenetic position of Myctophiformes, from mitogenomic evidence, in relation to basal neoteleostean orders and Acanthomorpha remains ambiguous. This is supported by comparison to previous mitogenomic studies [33,43,46,77] and nuclear genes [78]. Although we have included all neoteleostean orders in this study (Figure 4), this issue requires the inclusion of additional mitogenomes from all neoteleostean orders, and analyses carried out using newly developed models accounting for site-specific modulations of the amino acid replacement process, such as for example the CAT mixture model [79] implemented in phylobayes [80]. However, the latter program does not implement mixture of characters preventing the use of all information included in the mt genome, making this analysis premature and is therefore not employed in this study.

**Figure 5:**
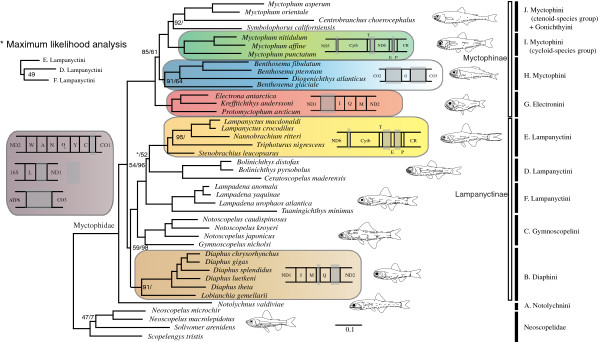
**Mitogenomic Myctophiformes phylogeny (123**_**n**_**RT**_**n**_**) rooted with Neoscopelidae.** Branch support from 1000 Bootstrap replicates and Bayesian posterior probabilities noted only if values are less than 100 – separated by a slash (/), respectively. One topological difference between the Bayesian- and ML-analyses is noted with bootstrap value included. Ten distinct myctophid lineages are noted (A-J) with respect to current tribal classification.

The myctophiform ingroup topology from the two datasets analyzed (87 taxa: 123_A_RT_n_ and 41 taxa: 123_n_RT_n_) showed differences related to lampanyctine tribal relationships (Figures 4 and 5). ML- and Bayesian analyses of the 87 taxa also showed incongruence concerning the phylogenetic position of *Triphoturus nigrescens* (Figure 4).

Neoscopelidae and Myctophidae were both recovered as monophyletic with *Scopenlengys tristis* recovered as the sister taxon to the remaining neoscopelids (low support) and *Notolychnus valdiviae* was recovered as the sister taxon to the remaining myctophids (high support). Myctophine and lampanyctine subfamilies were also recovered as monophyletic in all analyses. The tribe Diaphini was recovered as the sister lineage to the other lampanyctines, found in all analyses although with low support values, despite the unresolved tribal relationships within the remaining lampanyctines. The tribe Electronini was recovered as the sister lineage to the other myctophines with maximum levels of support and a clade consisting of *Benthosema* spp. and *Diogenichthys atlanticus* was resolved as the sister lineage to the tribes Gonichthyini and Myctophini. Ten myctophid lineages are recognized from this study and are noted according to current tribal classification as A-J in Figures 4 and 5. We note that deep myctophiform- and tribe-defining nodes were consistently found in all analyses, although support values indicate uncertainty concerning basal neoscopelid-, basal lampanyctine- and to a lesser degree basal myctophine phylogenetic relationships. Tribal relationships within Lampanyctinae (clades D, E and F) proved to be the most problematic part of myctophid phylogenetics from mitogenomics, showing incongruent results between datasets employed and mode of analysis (Figures 4 and 5).

### Diaphini tRNA-Met pseudogenes

**Figure 6:**
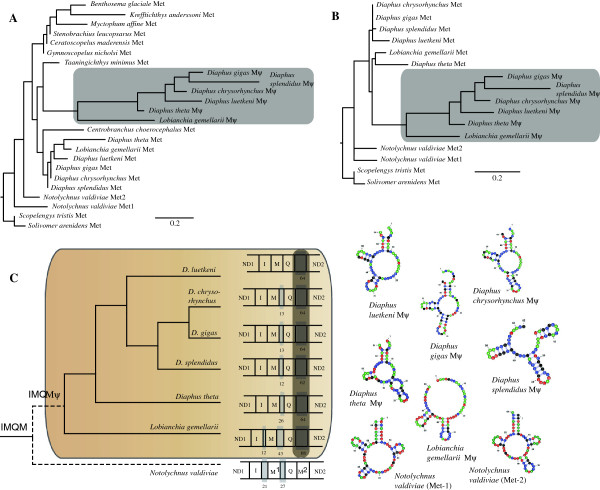
**K2P-distance analyses of tRNA-Met genes and the tRNA-Met pseudogenes found in Diaphini and *Notolychnus. *A**. Analysis done with randomly selected myctophid tRNA-Met genes using a neoscopelid outgroup. **B**. Analysis done with only diaphinid taxa and *Notolychnus* using a neoscopelid outgroup. Note the better phylogenetic structure in the pseudogenes compared to their counterpart tRNA-Met genes. **C**. Mitogenomic cladogram showing the IMQM_ψ_ – region in Diaphini. The truncated branch of *Notolychnus* signifies a phylogenetic position not corroborated from DNA sequences or tRNA-Met/tRNA-Met pseudogene distance analyses. Shaded areas represent INC-regions with number of base pairs noted for each taxon. Note the “gradual” removal of the INC-regions in diaphinid taxa except the tRNA-Met pseudogene (dark shaded). Secondary folding structures are presented for *Notolychnus* tRNA-Met1 and Met2 and all diaphinid tRNA-Met pseudogenes.

**Figure 7:**
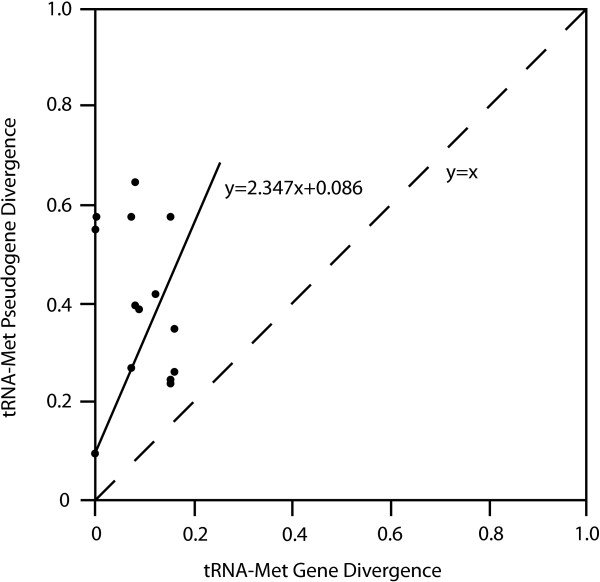
**Fifteen pairwise sequence divergences of tRNA-Met and tRNA-Met pseudogenes of the six diaphinid taxa included in this study.** Note the statistical significant higher sequence divergences in the tRNA-Met pseudogenes compared to the tRNA-met genes (*t*-test, df=13, P < 0.05).

All taxa within the tribe Diaphini show an IMQ-gene order in addition to an INC-region upstream from ND2 ranging from 62 to 68 base pairs. The INC-region shows varying degrees of sequence similarity to detected tRNA-Met genes with most tRNA secondary structures missing only the D- and variable loops of the typical tRNA gene cloverleaf secondary structure (Figure 6). Pairwise distances for tRNA-Met pseudogenes (INC-regions) were all much higher than for the detected tRNA-Met genes (Figure 7). In view of the IMQM_ψ_ gene order and putative tRNA-Met duplication in Diaphini, an interesting finding is observed in the monotypic basal branching taxon *Notolychnus valdiviae,* exhibiting two identified tRNA-Met genes resulting in an IMQM gene order. The two tRNA-Met genes both show the CAU-anticodon although they are highly divergent with respect to sequence similarity (~20% p-distance). Neigbour-joining distance method analyses of the tRNA-Met genes and their pseudogene counterparts showed clear phylogenetic structure in the diaphinid pseudogenes (Figure 6). Including the two tRNA-Met genes observed in *Notolychnus* (IMQM gene order) in the analyses, in order to examine a possible connection to Diaphini (IMQM_ψ_ gene order) concerning this gene-order rearrangement, showed no phylogenetic relationship between these taxa (Figure 6).

### Testing alternative phylogenetic hypotheses

Comparing constrained topologies according to morphology with the best scoring ML-tree from this study rejected a Notolychnini-Lampanyctinae relationship (*P* = 0.024; Table 2) as found from most morphological studies (Figure 2). A Notolychnini-Myctophinae relationship could not be rejected (*P* = 0.151) and the topology with Notolychnini constrained within Diaphini as indicated from the tRNA-Met duplication event was clearly not rejected (*P* = 0.407).

**Table 2:** Statistical comparisons between phylogenetic hypotheses using the AU-test (41 taxa Myctophiformes dataset)

**Topology**	**-ln L**	**Δ ln L**	AU*
(Notolychnus (Lampanyctinae, Myctophinae))	221112.283340	Best	0.733
((Notolychnus, Lampanyctinae) Myctophinae))	221130.952336	18.668996	0.024*
(Notolychnus, Myctophinae) Lampanyctinae)	221125.980254	13.696914	0.151
(((Notolychnus, Diaphini) Lampanyctinae) Myctophinae)	221118.943061	06.659721	0.407

*P < 0.05 = constrained topology rejected at the 5% confidence level.

## Discussion

### Monophyly of myctophiformes and the two families

This study recovered a monophyletic Myctophiformes and monophyletic families with Neoscopelidae as the sister family of the highly diversified Myctophidae (Figures 4 and 5; [3,5,81,82] although see Rosen [83]). We found *Scopelengys tristis* was a sister taxon to the remaining neoscopelids, including the monotypic endemic Sulu Sea taxon *Solivomer arenidens*[84], in congruence with morphology (Figure 2). Stiassny [5] presented characters in support for *Scopelengys* as sister taxa to the remaining neoscopelids, although she did note a possibility of apparently derived characters in *Neoscopelus* and *Solivomer* being secondarily reduced in *Scopelengys*. Analyses from the present study are congruent on this matter although support values are alarmingly low concerning the phylogenetic position of *Solivomer arenidens*. We found *Notolychnus valdiviae* to be the sister taxon to the remaining myctophids; however, morphological character states of *Notolychnus* such as dorsally located photophores, small larvae without pigmentation and a reduced otolith, have been extremely difficult to interpret concerning early myctophid divergence. Photophores are present in all myctophids except *Taaningichthys paurolychnus* and present only in one Neoscopelidae genus *Neoscopelus.* Our results show both of these genera nested within their respective families and the evolution of photophores within Myctophiformes is difficult to assess, i.e. whether photophores have evolved once or twice within ancestral myctophiforms. This question is pertinent from morphological considerations on photophore structure in Myctophidae and Neoscopelidae, as it is quite different in the two groups [85,86]. The phylogenetic positions of *Scopelengys*/*Solivomer* and *Notolychnus* in this study revives an interesting observation by Paxton [3] concerning parallel evolutionary trends for *Solivomer/*Lampanyctinae and for *Neoscopelus*/Myctophinae, regarding changes in jaw length and characters possibly associated with these changes. These characters have been difficult to interpret in terms of polarity and this study supports long jaw length as the plesiomorphic state in Neoscopelidae and Myctophidae from the basal branchings of *Scopelengys*/*Solivomer* and *Notolychnus*. Also, Stiassny [5] noted that important myctophiform character states like elongated jaw length and raised photophores show ambiguity concerning early myctophiform divergence.

A monophyletic Myctophidae was strongly supported by mitogenome sequences, a WANYC gene order and an INC-spacer between tRNA-Leu^UUR^ and ND1 (Figures 3 and 4). Rearrangement of the canonical vertebrate WANCY gene order was shared by all myctophids with the tRNA-Cys relocated downstream resulting in a synapomorphic WANYC gene order (Figure 5). A prolonged sequence (putative O_L_ + INC-sequence) in the canonical position of the putative origin of L-strand replication was found within all myctophids (see Additional file 4), although fishes within the tribe Electronini were difficult to determine due to additionally rearranged gene orders in this region (Figure 3). An INC-region between the tRNA-Leu^UUR^ and ND1 also represents synapomorphic evidence for Myctophidae (Figure 3). The size of this spacer ranged from 45–79 base pairs and we found no obvious tendency regarding size change for this INC-region (see Additional file 4). Neither the O_L_ + INC-region nor the Leu^UUR^/ND1 spacers showed sequence similarity in any species to any other mitochondrial genes. Neither could they be folded into clover shape secondary structures, so their origins are elusive. On the other hand, assembly of the INC-spacers showed clear sequence similarity within genera indicating the INC-regions to be inherited synapomorphic characters within the family. Smith et al. [87] reported a length expansion for a similar small INC-sequence located between tRNA-Val and 12S rRNA in a clade of serranid fairy basslet fishes (Serranidae). They showed the INC-sequence to be homologous for all genera from simultaneous analysis of nucleotides and gene order – a very difficult task for myctophids considering multiple gene order rearrangements and putative associated INC-sequences. The third myctophid synapomorphy from this study is an INC-region between ATP6 and CO3 ranging from 288 base pairs in *Notolychnus* to 9–43 base pairs in the remaining myctophids included (see Additional file 4). No conserved sequence motifs were found in this region. This is a peculiar spacer in the sense that the canonical vertebrate gene order for this region is ATP6/CO3 with no tRNA gene acting as punctuation mark for RNA processing.

### Monophyly of the two subfamilies and five tribes

The monophyly of the two subfamilies Lampanyctinae and Myctophinae was recovered from all analyses, although the low support values associated with this split are incomprehensible from morphological characters unambigously delimiting these two groups. As with the Myctophidae, the two subfamilies are fully supported by adult and larval morphology and no discussion is needed – character states are presented by Moser & Ahlstrom [28], Paxton [3] and Stiassny [5]. A well supported result from this study, differing from morphology, is the phylogenetic position of the tribe Diaphini within Lampanyctinae, resolved as sister tribe to the other lampanyctines. Adult and larval morphology within Lampanyctinae show incongruences and both hypotheses are different compared to our molecular results (Figures 2, 4 and 5).This shows problematic morphological characters, lampanyctine larval characters are noted as few and tentative in previous larval studies [30,88] and of little use above genus level, as well as molecular characters, no unique gene orders connect tribes and the mitogenomic phylogenetic sequence information is insufficient, as witnessed from the three different resolved Lampanyctinae topologies from this study (Figures 4 and 5).

Monophyly of the tribes Notolychnini (clade A in Figures 4 and 5), Diaphini (B), Gymnoscopelini (C), Lampanyctini (D+E+F), Electronini (G) and two subclades of Myctophini (H and I+J) are strongly supported from all mitogenomic DNA sequence analyses and various mt gene orders discussed below. Only the node delimiting the tribe Diaphini (B) show relatively low support values. The tribe Gonichthyini is tentatively placed from a single taxon as nested within a Myctophini subclade consisting of ctenoid-scaled species. Mitogenomic DNA sequence data from this study supports recognition of 10 mutually exclusive clades within Myctophidae (Figures 4 and 5).

### Phylogeny of tribes and genera

Students of lanternfish phylogenetics will note the position of *Notolychnus* (clade A) resolved as sister taxon to the remaining family Myctophidae from mitogenome sequences. Paxton [3] tentatively placed *Notolychnus* as sister taxon to the subfamily Lampanyctinae (Figure 2A). Subsequent parsimony analysis of the same data by Paxton et al. [30], including additional larval characters [27-29], placed *Notolychnus* in an unresolved Myctophinae-Notolychnini-Lampanyctinae trifurcation (Figure 2D). Subsequently, Stiassny [5] added four additional characters and reanalyzed the aforementioned data with a similar result, showing *Notolychnus* as the sister taxon to the remaining lampanyctines (Figure 2E). Most recently, Yamaguchi [24] scrutinized previously published characters (Figure 2A-E) once again and reanalyzed the data, resulting in a largely unresolved myctophid tree (Figure 2F). However, the position of *Notolychnus* was congruent with Paxton [3] and Stiassny [5]. The novel phylogenetic position of *Notolychnus* from this study, as the basal branching in Myctophidae, highlight the past issues concerning subfamilial affiliation and partly explains the lack of phylogenetically informative morphological characters in relation to the two subfamilies.

A monophyletic Diaphini (clade B) was well supported from both sequences and a unique IMQ-gene order (Figure 3), all in complete agreement with morphology, showing *Lobianchia* (2 species) as sister group of the very speciose *Diaphus* (78 species) [89-91]. Four species-groups of *Diaphus* have been suggested from the presence of Dn-Vn, Ant, Suo and So photophores (for photophore terminology, see Figure 1) [91]; however, our five species of *Diaphus* included were insufficient to validate anything on this matter. One very rare monotypic taxon *Idiolychnus urolampus*, missing from our study, was transferred from *Diaphus* to *Lobianchia* by Bolin [92] and most lately erected into its own genus *Idiolychnus* by the position of VO_3_ and possession of two SAOs [93].

Gymnoscopelini (clade C) sensu Paxton [3] was found to be monophyletic although not fully congruent with Ahlstrom et al. [88]. Unfortunately, the monotypic taxon *Lampanyctodes hectoris* is not included in this study, a myctophid showing ambiguous morphological characters concerning tribal placement. Paxton [3] noted *Lampanyctodes* as a possible early divergence based on several characters shared with the most plesiomorphic diaphinid genus *Lobianchia*. Conversely, Ahlstrom et al. [88] noted *Lampanyctodes* as being specialized within Gymnoscopelini from a similar larval form and development to that found within *Lampadena*, arguing that characters reflect habitat instead of true phylogeny.

*Ceratoscopelus* and *Bolinichthys* formed a clade (D) as either sister group to the remaining lampanyctines or included within them (Figures 4 and 5, respectively). Paxton [3] included these genera within Lampanyctini as closely related, whereas Ahlstrom et al. [88] placed them within Gymnoscopelini. Our study supports inclusion in Lampanyctini despite unresolved intrarelationships of this tribe. The clade itself is very distinct in terms of mt sequences and supported by both osteology [3] and larval development [30].

A tRNA-Glu gene could be detected in the canonical vertebrate position between ND6 and Cytb in all myctophids included in this study, except in a Lampanyctini subclade (E) comprising *Stenobrachius*, *Triphoturus*, *Nannobrachium* and *Lampanyctus* (Figures 3). In *Nannobrachium ritteri* and *Lampanyctus crocodilus,* we were able to determine a novel location of tRNA-Glu in the highly polymorphic region downstream Cytb including the tRNA-Thr and Pro genes, the CR and various INC-regions. Sequence similarities between tRNA-Glu from *N. ritteri* and *L. crocodilus* and the INC-regions between ND6 and Cytb were evident from sequence comparisons and putative secondary structures (see Additional file 3). Interestingly, this INC-region showed no sign of redundancy since all five taxa have retained a fragment equal in size to the typical length of tRNA genes (68–73 base pairs). We tentatively assign this gene order to the lampanyctine subclade E and note that this clade is congruent with Paxton [3] and Paxton et al. [30], although the latter notes that no synapomorphic character is present to actually define the clade. We expect this feature to be found within the unsampled genus *Parvilux*[94] as well. A new position of tRNA-Glu and retention of an INC-sequence between ND6 and Cytb has also been found in other vertebrates, e.g. the amphisbaenian reptile family Bipedidae [95], and seems to be one of the most common gene order rearrangements in vertebrates.

A clade consisting of *Lampadena* and *Taaningichthys* (F) is found monophyletic and completely congruent with morphology*. Taaningichthys* was previously a part of the genus *Lampadena*[4] although it was separated by Bolin [92] based on characters such as reduced number of photophores and flaccid body structure. Nafpaktitis and Paxton [96] noted that *Lampadena chavesi*, *L. dea* and *L. speculigera* were more closely related to *Taaningichthys* than the remaining species of *Lampadena* based on expanded neural arches on the anterior vertebrae as found in *Taaningichthys*. In fact, Paxton [3] noted that discriminating characters between the two genera were hard to find. *Lampadena* and especially *Taaningichthys* are among the largest and deepest living myctophids, with the latter showing reduction of the lateral line and in the number of body photophores. This apparent reductional trend has resulted in one taxon, *Taaningichthys paurolychnus*, having lost all body photophores although retained the supra- and infracaudal glands. *Lampadena yaquinae* is found nested within the *Lampadena* species included and corroborates Paxton [97] in synonymizing *Dorsadena*[98] with *Lampadena*. Likewise, the subgenera *Lampadena* and *Lychnophora* proposed by Fraser-Brunner [4], supported by only an elevated PO_4_[96], are not supported from this study. Increased mitogenomic taxon sampling of these two genera is interesting concerning evolution of photophores within these deep dwelling genera showing atypical character states within the Myctophidae [99].

The tribe Electronini (G), erected from low-leveled PVO and PLO photophores [25], composes a subset of the tribe Myctophini as recognized by Paxton [3]. Our results clearly support Electronini as a separate tribe [100] from both DNA sequences and long (>200 base pairs) synapomorphic INC-regions upstream the tRNA-Ile gene (Figures 3 and 5). We recovered a sister relationship of Electronini to the rest of Myctophinae, a result also presented by Paxton et al. [30], although cladistic analyses of morphological characters failed to recover this result (Figures 2E-F).

The IQM-regions of species included in the genera *Benthosema* and *Diogenichthys* (clade H) were peculiar and the IQM-region and WANYC-region of both Electronini and *Benthosema-Diogenichthys* were highly differentiated compared to all other myctophids. *Benthosema glaciale* showed new positions of tRNA-Gln and -Met to the downstream region of the WANYC-region (Figure 3) representing a novel gene order in vertebrate mitogenomes. *Diogenichthys atlanticus* showed an IQMM gene order and the canonical vertebrate gene order was observed within *Benthosema pterotum* and *B. fibulatum*. Taxon sampling prevents us from discussing rearrangements for these taxa at this point; however, a close relationship between the two genera is consistent with morphology. *Diogenichthys* was separated from *Benthosema* by ventrally leveled Prc 1 and 2 in addition to various jaw differences and hooked dentary teeth, a character state only found within lampanyctines. Similar to *Notolychnus, D. atlanticus* is a diminutive species with adult maximum size < 30 millimeters and could represent a problematic branch within this clade considering character states related to miniaturization. Diminutive species are also found within the genus *Diaphus*. The *Benthosema*-*Diogenichthys* clade was supported from DNA sequences and an INC-region up- or downstream tRNA-Gly; all three species of *Benthosema* showed an INC-region flanked by an upstream CO3 gene and downstream tRNA-Gly. In addition, *B. glaciale* showed an INC-region after tRNA-Gly. In *D. atlanticus*, however, the INC-spacer was located downstream from the tRNA-Gly gene (Figure 3). Interestingly, two tRNA-Gly genes separated by a large INC-region were observed in this same region in *Electrona antarctica*, although none of the INC-regions could be shown to have any sequence similarity to each other or to tRNA-Gly. We have assumed an INC-sequence associated with tRNA-Gly as a character state for the *Benthosema-Diogenichthys* clade, although with reservations. In view of *E. antarctica* possesing two tRNA-Gly genes we note that an association of this region in *E. antarctica*, *D. atlanticus* and *Benthosema* spp. is plausible and should be elucidated with increased mitogenomic taxon sampling.

The tribe Myctophini was not supported from this study as the sister clade to Electronini, because the single representative of the tribe Gonichthyini (*Centrobranchus choerocephalus*) included in this study renders Myctophini paraphyletic. We note that broader taxonomic representation of the genera *Gonichthys, Centrobranchus, Tarletonbeania* and *Loweina* is necessary concerning phylogenetic position of Gonichthyini within Myctophinae. However, evidence from mt sequences and a unique gene order in part of Myctophini (Figure 5) is consistent with the results from Paxton [3] stating that”..*A number of characters suggest that the* Myctophum-Symbolophorus *line gave rise to the slendertails* [ed. tribe Gonichthyini]..”. Additional evidence for this result comes from the non-monophyly of Gonichthyini based on larval characters [27] (Figure 2C) and the analyses by Stiassny [5] and Yamaguchi [24] (Figure 2E-F) showing *Symbolophorus* a basal branching within Gonichthyini. This study confidently resolves the genus *Myctophum* into two different clades from mitogenome sequences and also from a unique gene order involving ND6, tRNA-Glu and Cytb (Figures 3, 5). *Myctophum affine*, *M. punctatum* and *M. nitidulum* (clade I) showed this particular gene arrangement whereas *M. asperum* and *M. orientale* showed the typical myctophid gene order also found in *Centrobranchus choerocephalus* and *Symbolophorus californiensis*, the latter four forming a well supported clade J (Figures 4 and 5). Two species-groups are currently recognized in the genus *Myctophum* based on cycloid versus ctenoid scales [101] corresponding to the two separated groups of *Myctophum* found in this study. Moser and Ahlstrom [27] discussed eye shape within *Myctophum* and noted this genus as difficult to distinguish phylogenetically, with stalked eyes usually absent, although present in e.g. *Myctophum nitidulum* and *M. punctatum* corresponding to the cycloid-scaled *Myctophum* lineage from this study. This result is, however, complicated by stalked eyes also being found in all species of *Symbolophorus*[27]. Clearly, mitogenome sequences and a unique gene order in the cycloid-scaled species of *Myctophum* strongly suggest that the two groups are in fact different lineages within Myctophini and that the tribe Gonichthyini is nested within the ctenoid-scaled group. This indicates ambiguous relationships from adult and larval morphology or convergent evolution within Myctophini lineages. Paxton [3] noted that no additional characters are present within *Myctophum* to support a split between cycloid- and ctenoid-scaled *Myctophum* species. The term”near-surface” myctophids has been used for a group of myctophids represented within the genera *Loweina*, *Tarletonbeania*, *Gonichthys*, *Centrobranchus*, *Symbolophorus* and *Myctophum* with most species showing DVM behaviour and most being easily netted in surface layers at night time [102]. Denser taxon sampling within Gonichthyini, and the ctenoid-scaled Myctophini group as recognized from this study, should clarify if DVM patterns and phylogeny are correlated.

### Diaphini tRNA-Met pseudogenes

Our included taxa in the tribe Diaphini opens up for a unique empirical case of a putative duplication event that can be observed through time for retention and deletion of various genes/pseudogenes in separate diaphinid taxa. All tRNA-Met pseudogenes were retained with 64–68 base pairs whereas other INC-regions between the tRNA genes in this segment (grey areas in Figure 6) seem to be in the process of being removed. Phylogenetic congruence between the mitogenomic sequences and the diaphinid pseudogenes (Figures 5 and 6, respectively) is clear despite the short pseudogene sequence lengths. Only one other vertebrate group, scarrid parrotfishes, shows a similar pattern of gene rearrangement of the IQM-region and retention of the pseudogene [103], although the pattern of retention and deletion of INC-regions in parrotfishes is much less pronounced (see Additional file 5). We note that the tribe Electronini and other taxa within the subfamily Myctophinae exhibit gene rearrangements, albeit different in organization, associated with this particular region (Figure 3, see Additional file 4). We acknowledge the missing discussion of this region in the subfamily Myctophinae; however, our results concerning this particular region in Myctophinae awaits increased taxon sampling. Topology testing using the AU-test could not reject a Notolychnini-Diaphini relationship (*P*=0.407; Table 2) in contrast to morphology, mitogenomic sequences and our NJ-analyses of the INC-regions (Figures 2, 5 and 6, respectively).

We note a third case of various anuran putative duplication events of the tRNA-Met gene [104] and although this case is reminiscent of the low sequence similarity between the two detected tRNA-Met genes as seen in *Notolychnus*, the tRNA-Met anticodon is repeatedly detected in anurans making this situation different to lantern- and parrotfishes.

### Mt gene order rearrangements in vertebrates

Observations of unique gene rearrangements in Myctophidae, combined with their utility as clade defining synapomorphies, are quite numerous compared to similar findings within other vertebrate groups [105]. It should be noted that whereas mt sequences and gene order rearrangements resolve myctophiform relationships in a highly corroborative manner in this study (Figure 5), taxon sampling and unknown selection on gene order and INC-sequences are problematic when inferring phylogeny. Clearly, gene order rearrangement hotspots are present in vertebrate mt genomes and selective constraints could plausibly show convergent evolution to be the reason in some cases [106]. Almost all gene order rearrangements and INC-regions presented for Myctophidae have also been found within other vertebrate groups. For example, the INC-sequence present between ATP6 and CO3 is found within *Gonostoma gracile* (order Stomiiformes) [45] and in various deep-sea anglerfishes [47]. Birds (class Aves) all have a clade-defining rearranged gene order encompassing a fragment ranging from ND6 to the CR [107], although multiple variations have subsequently occurred on top of this synapomorphy [40]. Plethodontid salamanders show multiple gene rearrangements with likely concerted evolution on duplicated regions [108]. The latter also suggested an INC-sequence between tRNA-Thr and -Pro possibly functioning as an alternative initiation site for replication. All myctophiforms successfully determined for this mt site show an INC-sequence between tRNA-Thr and -Pro, although the length of this spacer in myctophids is highly variable (see Additional file 4). Bakke et al. [109] postulated a selective advantage as a protein-binding site for this spacer, supported by a conservative motif sequence in codfishes. Concerted evolution on CR-duplicates is also reported from snakes [110] and mantellid frogs [111], with the latter strongly indicating recombination associated with the CR in various taxa exhibiting duplication events. Varanids and monitors show events in the variable region upstream of the CR [112] and acrodont lizards show a QIM gene order [113]. As mentioned, scarid parrotfishes all have an IMQ-gene order showing similar patterns as found in Diaphini, with an INC-sequence (pseudogene) retained upstream the ND2 gene from a putative duplication of tRNA-Met [103]. The latter suggested that the tRNA-Met pseudogene neighbouring ND2 retained a function as punctuation mark for ND2 mRNA processing. Mt tRNA transcriptional efficiency in relation to distance of initiation of replication was explored by Satoh et al. [114], who found support for highly expressed genes positioned closer to the control region. Selection on INC-regions in relation to bordering genes is an important theoretical caveat when using INC-regions as phylogenetic markers. Satoh et al. [48] initiated mt gene order comparisons within codfishes (Gadiformes), showing rearranged gene orders and INC-regions in a possible similar fashion to Myctophidae. Most notably one species, *Bathygadus antrodes,* possessed all of the gadiform rearrangements although somewhat differently arranged from other subclades. This indicates early rearrangement events subsequently modified into presently observed synapomorphic gene orders for specific gadiform clades. The most basal branching in Myctophidae, *Notolychnus*, shows mt rearrangements such as two tRNA-Met genes and a comparatively large INC-sequence between ATP6 and CO3. These features are either modified or lost in other myctophids. On the other hand, several rearranged gene orders found in various myctophid tribes are not traceable in *Notolychnus.* Gene order rearrangements within Myctophidae provide a unique example of the phylogenetic utility of mt gene orders and the problems associated with this group of markers.

## Conclusions

Mitogenomes from myctophiform fishes provide a unique empirical case of mt DNA sequences and gene order rearrangements that corroborate evolutionary history. Future mitogenomic determination of the 200+ remaining species within Myctophidae will most certaninly increase our knowledge concerning issues associated with gene order rearrangement events and their utility as phylogenetic markers. Multiple different gene orders found throughout the mt genome, utility as phylogenetic markers and the unknown causes and mechanisms associated with gene duplication and subsequent retention and/or removal of sequences, calls for the determination of complete mt genomes over single genes. Our results support the use of osteology and photophores in phylogeny of Myctophiformes, the latter being a very unique feature of any deep-sea group of organisms. Larval ontogeny is also informative albeit difficult to use on higher phylogenetic levels and particularly within the subfamily Lampanyctinae.

## Competing interests

The authors declare that they have no competing interests.

## Authors’ contributions

All authors contributed in preparation of the study. JYP performed molecular lab work, initial analyses and drafted the original manuscript. DR, HT and TPS continually reviewed and discussed lab-procedures. JYP and EW finalized the analyses. JYP, IB, EW and MM finalized the manuscript. All authors read and approved the final manuscript.

## Supplementary Material

Additional file 1 **Table T2.** Partitioning schemes used in the present study suggested by PartitionFinder. Click here for file

Additional file 2 **Saturation plots of pairwise distances of 1st, 2nd and 3rd codon positions in the 12 protein coding genes employed in the analyses.** Vertical axis shows p-distance for each codon position against total p-distance (horizontal axis). Saturation from RY-coding of the 3rd codon positions included (only transversions valued) although not used in the present study from early onset of saturation. Click here for file

Additional file 3: Figure S1 Secondary structure of INC-regions. Sequence alignments and secondary tRNA structures of INC-regions are shown for synapomorphic spacers pertaining to gene order rearrangements 1, 3, 5 and 8 presented in Figure 3. The remaining INC-regions presented in Figure 3 are relatively short sequences and are not included. Click here for file

Additional file 4: Table T1 Base pair details of INC-regions found in Myctophidae. Eight mt regions correspond to gene order and INC-region synapomorphies presented in Figure 3 with slash (/) denoting bordering genes. The last column includes mt information from the polymorphic region from tRNA-Thr and downstream towards the control region (CR). Numbers of INC-sequence base pairs are parenthesized next to flanking genes. Greater-than sign (>) and hyphen (−) denote partial and no sequence, respectively, from failure to complete the sequences. Click here for file

Additional file 5 **IMQM**_**ψ**_** gene order in a clade of scarid parrotfishes.** Taxa constituting a monophyletic clade in Scaridae all show similar patterns in the IMQ-region as observed within Diaphini. Grey areas show INC-regions with number of base pairs noted for each taxon and the arrow indicates possible gradual removal of INC-regions. Click here for file
