# Supplementary material for: Mitogenomic sequences and evidence from unique gene rearrangements corroborate evolutionary relationships of myctophiformes (Neoteleostei)
Source: BMC Evol Biol. 2013 Jun 3;13:111. doi: 10.1186/1471-2148-13-111 (PMC3682873; doi:10.1186/1471-2148-13-111)
Supplement: Additional file 1 — Table T2. Partitioning schemes used in the present study suggested by PartitionFinder. [file 1471-2148-13-111-S1.pdf]

Six partitions used for 87 taxa: $123_A RT_n$

1	2	3	4	5	6
**CO1**	ATP6	ND2	ATP8	12S	22 tRNAs
**CO2**	ND1			16S	
**CO3**	ND3				
**Cytb**	ND4				
	ND4L				
	ND5				

Six partitions used for 41 taxa: $123_n RT_n$

1	2	3	4	5	6
**ATP6**	CO1	ND1	ND2	12S	22 tRNAs
**ATP8**		ND3		16S	
**CO2**		ND4			
**CO3**		ND5			
**Cytb**					
**ND4L**					
