# Supplementary figures and images for: Mitogenomic sequences and evidence from unique gene rearrangements corroborate evolutionary relationships of myctophiformes (Neoteleostei)
Source: BMC Evol Biol. 2013 Jun 3;13:111. doi: 10.1186/1471-2148-13-111 (PMC3682873; doi:10.1186/1471-2148-13-111)

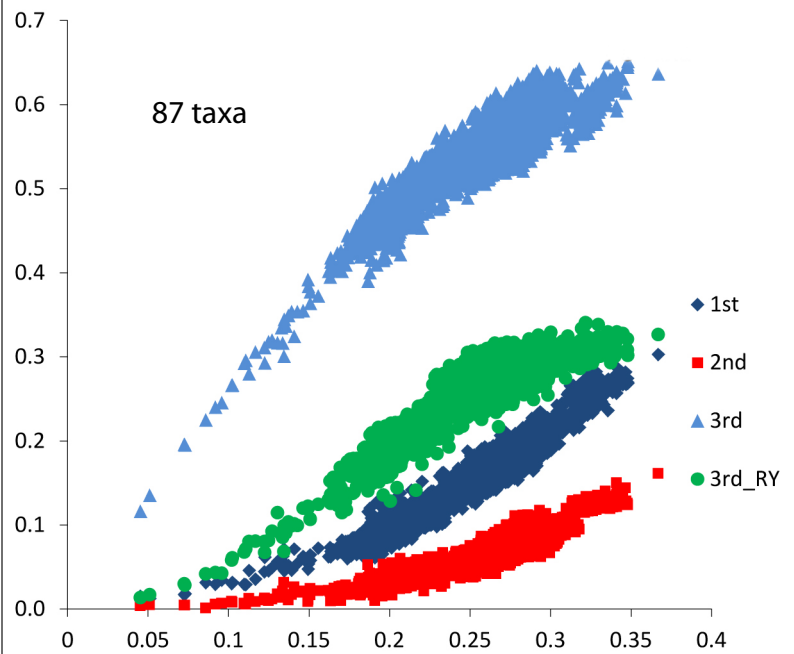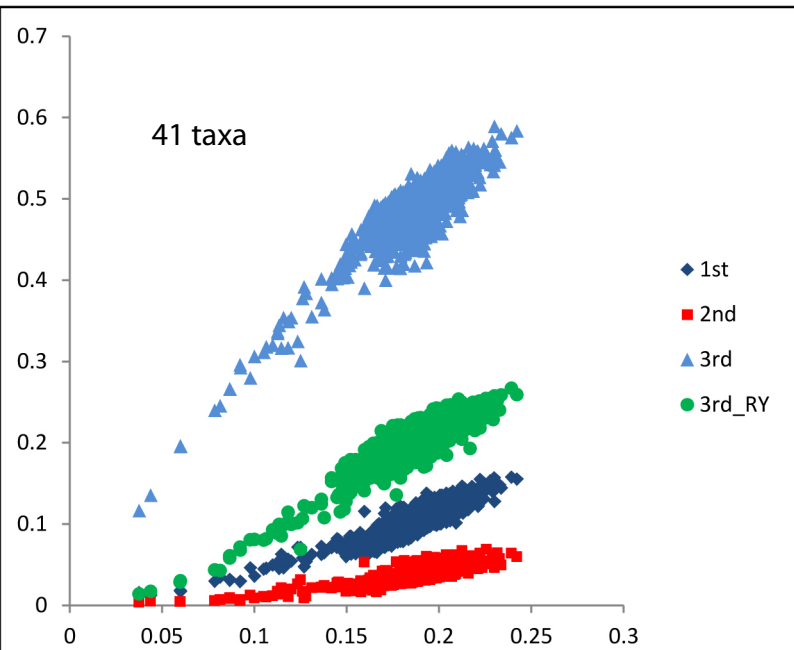

Supplement: Additional file 2 — Saturation plots of pairwise distances of 1st, 2nd and 3rd codon positions in the 12 protein coding genes employed in the analyses. Vertical axis shows p-distance for each codon position against total p-distance (horizontal axis). Saturation from RY-coding of the 3rd codon positions included (only transversions valued) although not used in the present study from early onset of saturation. [file 1471-2148-13-111-S2.pdf]
