# Supplementary material for: Mitogenomic sequences and evidence from unique gene rearrangements corroborate evolutionary relationships of myctophiformes (Neoteleostei)
Source: BMC Evol Biol. 2013 Jun 3;13:111. doi: 10.1186/1471-2148-13-111 (PMC3682873; doi:10.1186/1471-2148-13-111)

LocARNA-alignments of INC-regions in Myctophidae. Parenthesized numbers refer to the gene order rearrangement involved (presented in Figure 3).

Myctophidae (1). CLUSTAL W --- LocARNA 1.5.2 - Local Alignment of RNA

```
                   .............................---------------......................................-------------
Benthosema_glaci TACGCCCCCCCCACCCC-----GCCGTTG---------------GGGGGGTGA-GGCG-----GGGGGGGGGGTCGTCACTA-------------
Krefftichthys_an TTTCCCCCGCCC--------------TTG---------------GGGCCCCCA----------TAATTACTAGACAGTTCT--------------
Lobianchia_gemel GGACCCCCCCCACCGGGGGGAAGGGGGGG---------------GGGGCCCCCCCCCGAAAAATGTGGGGGGGGTTTGAAATA------------
Symbolophorus_ca ATCGCCCCCCCC-CCGC-----CCTAAAA---------------GATCAAGGC-GGGG-----GGGGGGGGGCACCTACATG------------
Benthosema_fibul -----CACCCCCCCCCGCCGTTATACAAAAA-------GGGG--GGGGGGGGGGGG-GGAATCTAAAGC----TTCT--CCTTTGC---------
Benthosema_ptero --CGGCCCC-CCCCCCGCCGAAAATTAAAAAGGGGGGGGGAAGGGGGGGGGGGGGGGGGGGGGGAAGAATCGGGGATTCTGCGGGGC---------
Bolinichthys_dis CGTTCCCCCTCGCCGG--GGTGGGCAAGGTCGAGGGGGAA--CCCGGCAATGCCGGGGCAGCTCTGCTATCGTGTAAGC-TCTTCCTAC---GTA
Bolinichthys_pyr CTTTCGCCCTCGCCGG--GGTGGGCAAGGTCGAGGGGGAA--CCCGGCAATGCCGGGGCAGCTCTGCTATCGTGCGAGC-TCTTCCTAC---GTA
Centrobranchus_c CGTTCCCCCTCGCCGG--GGTGGGCAAGGTCGAGGGGGAA--CCCGGCACTGCCGGGACAGAAAAAAAAAAAAAAAACTCCTTCTAGAG---GCA
Ceratoscopelus_m CGTTCCCCCTCGCCGG--GGGGGGCAAGGTCGAGGGGGAA--CCCGGCGGGGCCGGGGCAGCTCTGGTCTCAGGAAACTCAAGGCCTAC---GTA
Diaphus_chrysorh -GTCTCCCCCGCCATTGGGAATGGCGA-G-GCGGGGGAGACCCGGGTAA-AACCCGGAGTACCTCTGAGGTGGGCG-AACCACCTTAAG---A-T
Diaphus_gigas -GTCTCCCCCGCCATTGGGAATGGCGA-G-GCGGGGGAGACCCGGGTAA-AACCCGGAGTACCTCTGAGGTGGGTGCGCCCCCCTCAAG---A-T
Diaphus_luetkeni CGTCTCCCCCGCCCTTGAGGGGGGGAA-G-GCGGGGGAGACCCGGGTAATAACCCGGGGTACCTCTGGGTGGGAAGCAACCCTATAAGG---T--
Diaphus_theta CGTCTCCCCCGCCGTTCAGGACGGCGA-G-GCGGGGGAGACCCGGGTAACAACCCGGGGCATATCTGCGTGTAAACTATCTTTTTTTAG---GGC
Diphus_splendidu -GTCTCCCCCGCCATTAGAAATGGCGA-G-GCGGGGGAGACCCGGGTAA-AACCCGGGGTACCTCT-GGGGTAACGGAAACCCTCTAAG---G-C
Electrona_antarc -----TACCCCTCCGCCCTCTCGGGGGGGGGGGGGCGAAA--CGAGAACAAACCCCGGTGATCGCCGGGTGCTTCGCACTCTCC-----------
Gymnoscopelus_ni CCTTCCCCCCGCCGTTC-GGGCGGCGAG--GCGGGGGGAAAGCCGGTCA---CCCGGAGTCCCTCTAATTCTCAGGAGGTTAAGTCTCC--TAGT
Lampadena_U_atla C-GTTTCCCCTCGCCAGAGGCGAGCGAGGCGAGGGGAAAC--CCCGGGCACTTCCGAGGGAGTGCCTGCTGTCCCA---CCCGCTG---------
Lampadena_anomal CGTTTCCCCTCGCCAGA-GGCGGGCGAGG-CGAGGGGAAACCCCGGGCACGACCCGGGGGCCACTCTGGTCTGGCTCTGCCCCGCTTTC----TA
Lampadena_yaquin CGTTTCCCCTCGCCAGA-GGCGGGCGAGG-CGAGGGGAAACCCCGGGCACAACCCGGGGGCAGGTCTGGTCTGGCTCTGCCCCGCTTTC----TA
Lampanyctus_intr CGTTCTCCCTCGCCGG--GAGGGGCAAGG-CGAGGGAGAA--CCCGGCACGGCCGGG-GAATCTCTCAGGTCAGGCGCGCCTGGTTCAC---GTG
Lampanyctus_macd CGTTCTCCCTCGCCGG--GAGGGGCAAGG-CGCGGGAGAA--CCCGGCACGGCCGGG-GAATCTCTCAGGTCAGGCGCGCCTGATCCAC---GTG
Myctophum_asperu CACCCCCCCCCGCCTTTTAAACGTCGAGGGGGGGGGGGGGG--GGGGGAACAGCCTGAGGGTTGTTGGGCCTTCTAAAATCTGAAGGCT-------
Myctophum_nitidu TACATTTGCACCGCCCCCCCCCCCCCCCCAAAGGGGAGGGGAAGGGGGGGGGGGACGGCAACTGAGC-CTGGCTGCTC-TGTAAG---------
Myctophum_orient -----TTCCCCTCCCCCTCCGCGGGCGTCGAGGCGGGGGAGG--GGGGGGGGAAACAACTTCCCCTCCGGGGGAAGTCAGTAAGTG---------
Myctophum_puncta -----AACGCCCCCCCCCCCCGCCGTTAAGGC-------GGGC--GAGGCGGGGGGG-GGCCGGTAGCCACC-TGGCG--TTGCTCG---------
Nannobrachium_ri -GTTCTCCCTCGCCGG--GAGGGGCAAGG-CGAGGGAGAACACCCGGCACGGCCGGGGGAATCCCTGATGACGAGGGAGGCCGGTCCGC---GAG
Notolychnus_vald ------GGTTCCCCCCGCCGGGGGCCGGCAA-------GGCG--GGGGGAACCCCC-CCCCCCCTTTTGG-GGGGG--GGGGTCT---------
Notoscopelus_cau CGTCTCCCCCGCCGTTCGGGCCGGCGA-G-GCGGGGAAGACCCCGG--AGAACCGGGGATATCTCTGGGTGGGAAGGAAACCCTCCAGG---A--
Notoscopelus_jap CGTCTCCCCCGCCGTTCAGGACGGCGA-G-GCGGGGGAGACCCGGGTAACAACCCGGGGCATATCTGCGTGTAAACTATCTTTTTTTAG---G--
Stenobrachius_le CGTTCTCCCTCGCCCTACGGGGAGCGAGG-CGAGGGAGAACCCCGGTCACAGCCGGGGGAATCCCCAATATCTAGGGAATCCGGTCCAC----GA
Taaningichthys_m AGTTTCCCCTCACCCTTAAG-GGGCGAGG-TGAGGGGAAACCCCGAGCTCGCTCGGGGGTACCTCTGGTCTCGTGGGAAGTCGACTCACCGCGAA
Triphoturus_nigr AATTCCCCGAGCCATCACTTTTACTGGGCAAAAAAAAAAAAAAAAGGGGGGGGCGTAGCCCCAGAAGTCTGGGGTCGTGGGAAGTCGACTTAGAGG
                 ...............................................................................................
```

Lampanyctini subclade (3). CLUSTAL W --- LocARNA 1.5.2 - Local Alignment of RNA

```
                 ...((((((...(((...)))))))))).......((............))................)).......
Lampanyctus_intr AGTTCTTCCCCAGCTTCCGCCAAGATCCG-TGACTAATAAACCACCGTTGTGACCCGACTACAAGAACA-ATA
Lampanyctus_macd AGTTCTTCCCCAGCTTCCGCCAAGATCCG-TGACTAATAAACCACCGTTATGACCCGACTACAAGAACA-ATA
Nannobrachium_ri GGCTCTCCCCCCGCTCCCGCCGGGACTCG-TGACTAATAAACCACTGTTGTGACCCAACTACAAGAACA-ATA
Stenobrachius_le AATTCTTGCCCAGTTTTAAACAGAGACCGATGACTCAAAAACCACCGTTGTAACTCAACTACAGGAACAGTTA
Triphoturus_nigr -GATTCTTGCCTCGGCTTCCGCAAGATTG--TGTGATTTAA--ACCACCGTGATACAACTACAAAAACAGTTA
                 -..((((((...(((...)))))))))....--.((......--...))................)).......
```

Myctophidae (5). CLUSTAL W --- LocARNA 1.5.2 - Local Alignment of RNA

```
                  ...........((((((......................................((((....)))))))).))))...
Benthosema_fibul CTC-------CGCTG-AGCTCACCACT------------ATTTAGCATAGAATA--AAGGTTAAAACCCGCCCCCAGC--A
Benthosema_glaci AACC---CGTTGGGCCTTGACCAGCTC------------ACCTATCAGTGACATA-AGGATGACACCTTCTCCCCAAC--A
Benthosema_ptero CCT-----GCTGGCCCCCCAACATTTT------------AT--A------------AAGGTTAAAACCTGCCCCCAGC---
Bolinichthys_dis C---AGGCCATGGTGCCAC---ACCCAG-CCGG-----CTTCTGCCCGAACATAAGGAGGCTGAACCCTCCC--CCTGGCA
Bolinichthys_pyr C---ATGCCATGGTACCGC---ACCTAA-CCGG-----CTTCCGCCCGAACACCAGGAGACTGAGCCCTCCC--CCTGGCA
Centrobranchus_c CTC-----TCCGGCCCAGTATCGGCCA------------TCAAAA-----------GAGGGTGGCACCTCTGCCCGGTA-A
Ceratoscopelus_m AAT-----GCCACCGCCACAGCCGATT------------TCCCCTCCGGCTC-A--GAGATTGGATCCTCCCCTTGGC--A
Diaphus_chrysorh -CC----CGCCAGCCCCATAAAGCAGT------------AC-ACTAATCAAACTC-GAGATTAATCTCTCCGCCTGGC--A
Diaphus_gigas -CC----CGCCAGCCCCGTAAAACAGT------------AC-CCTACTCAAACTC-GAGATTAACCTCTCCACCTGGC--A
Diaphus_luetkeni -CCC---CGCCAGTCCTTCAAAACAAT------------GCAGCCCCTCAACCTC-GAGATTAATTTCTTCCCCTGGC--A
Diaphus_splendid -CCT---CGCCAGCCGCACAAAGCAAT------------GCACCTCCCCCAAC-C-GAGATTAACTTCTCCACCTGGC--A
Diaphus_theta -CCT---CGCCAGCCCCACAAAACGAT------------TTGTCCCACAAAACTC-GAGATTGGCTTCTCCGCCTGGC--A
Diogenichthys_at CCC-----GCTAGGAACCCACCCCCCA------------AC--AC-----------GAGGCTAAAACCTCACCCTAGC--A
Electrona_antarc AGT-----GCCCACGCCAGA---CGCC------------GCCC-CCTGTCAC----GAGGGTAAAACCTCTCGCCGGT--A
Gymnoscopelus_ni TAA-ACGTCATGTGGTCTTAGGACCTAGCCCGG-----CTGCCGCCCAGGCCCAAGGAGGTTAAAGCCTCCT--CCTGGCT
Krefftichthys_an AAA-----GCCTACGCCAAA---GACC------------ACGCAGCCGTCAC----GGGGGTAAGACCTCTCGCCGGC--A
Lampadena_U_atla CGACACGCCACATA-CCCTCAAACCCGG-CCGG-----TTACTACCCGGACAGGAGGAGACTAAAACCTCCA--ACTGGCA
Lampadena_anomal CGACACGCCCCATACCCCCTGGACCCAG-CCGG-----CCACCGTCCGGACAAAGGGAGGCTTAAACCTCCA--TCTGGCA
Lampadena_yaquin CGACACGCCACATGCCCCCTAAGCGCAG-CCGG-----CTACCGCCCGGACAAAGGGAGGCTTAAACCTCCG--TCTGGCA
Lampanyctus_intr T---ACGCCATACAACCCT-AAAC-TCGAGC-G-----TCCTCGCCTGACCGCACTGAGGGTAAAACCTCTC--ATTGGCA
Lampanyctus_macd T---ACGCCACACAACCCC-AAAC-TCGAGCAG-----CCCTCGCCTGACCGCACTGAGGGTGAAGCCTCTA--ATTGGCA
Lobianchia_gemel CCCCACCAGCATGGCCCTAAACAAACT------------GGCTTACCGCC-CTTCAGAGAGTGAAACCTCCCACTGGC--A
Myctophum_affine AC------GCTAGATTATACAGCCGAT------------TATAAAGTGCGTAA---GAGGTAAAACTCTCGCTAAGCA--A
Myctophum_asperu C-T------CCGCCGGACTAATAAACA------------AC---------------GAGGATAGCCCCTCCCCCCGGC--T
Myctophum_nitidu TC-------CACTAAATGTGA-CGACC------------ACAAAAGCACATAA---GAGGTAAATC-CTCCCCAAGCA--A
Myctophum_orient CTT------CCGCCGGGAATTACTTCG------------AT--TA-----------GAGGATAATCCCTCCCCCCGGC--T
Myctophum_puncta TCC-----CGCCCCGGAGC--CCGTCA------------GCCATACCAGCACAA--AAGGGTCAAACCTTCCCCGGCG--A
Nannobrachium_ri C---ACGCCATATGTTTCC-AAGATTCGATGGG-----CCCTCGCCCGACCATACTGAGGGTAAGACCTCTA--ATTGGCA
Notolychnus_vald CACCACAAGCTAGTGCACAAA-----A------------CCTATGCCAGGACAGAGGAGACTAAACCCTCCCACTGGC--A
Notoscopelus_cau TAG-ACGTCAGTGGCCCATTATTAAATTACTAGCCCCATTGCCGCCCGGACT-AAGGAGTATAAAACCTCCACCCCTGAAC
Notoscopelus_jap TAA-ATGTCAGTGACCCAGTACTACTCAACCAG-----TTGCCGCCTGGACTAAAGGAATATGAAACCTCCACCCCTGATT
Notoscopelus_kro TAA-ACATCAGTGGCCTAATAATTTTATCCC--------TACTGTCCGGGCTAAAGGAGTCTAAAGCCTCCACCCCTGAAT
Protomyctophum_a CCG-----CCCACACAGACACCACACG------------TC--AC-----------GGGGTTAAAGCCCCTCACCGGC--A
Stenobrachius_le C---ACGCCATATAACCCT-GAACATCGGCCGA-----CTTCTGTCCGGCCTCACCGAGGGTAAGACCTCCA--ACTGGCA
Symbolophorus_ca TCC-----TACGCCG-AGCCGACCATG------------AAT-----ACAACAA--GAGGGTAAAACCTCGCCCCGGC--A
Taaningichthys_m CGC-----CCCACTTCATTTACGGGC-------------AC--AC-----------GAGGCTGAAACCTCGCCAAAG----
Triphoturus_nigr ----------CGCCATTGAAACCTTA------------------------------GAGGGCACCCCCTCCAATTGGCA-G
                 ----------.((((((.......-------------------------------((((....)))))))).)))).-.
```

Myctophinini subclade (8). CLUSTAL W --- LocARNA 1.5.2 - Local Alignment of RNA

```
                   ----------......(((......)))....---------((((...--...)))).................---------...((....))......
Benthosema_fibul   ----------AAACTAGCCCTAAAACGCTTGA---------CCCGACT--AATCCCCGA-----AAAACCGCCC---------CCTCTCCCCGCA-----
Benthosema_glaci   ----------ACCTCAGCGTCGCAACTCCTTAG--------GCCGACA--ACTCGGCAATACAGAAAATGACCC---------CTAGGGCCACCACCAAC
Benthosema_ptero   ----------ATACCGGCTATCAATGGCC-GA----------TCCGGCT--AACC-CCAA-----AAAACCACCC--------------------------
Diogenichthys_at   CTTTAACCTTACAACACGGACTTAACCGACCAAAACGTATCCCTTATATTAACAAGGGACTTAATCACCTACCCCAACTAAGCCGTGGGACCATGCGCA
                   ................(((......)))...............((((........)))).............................((....))......
```

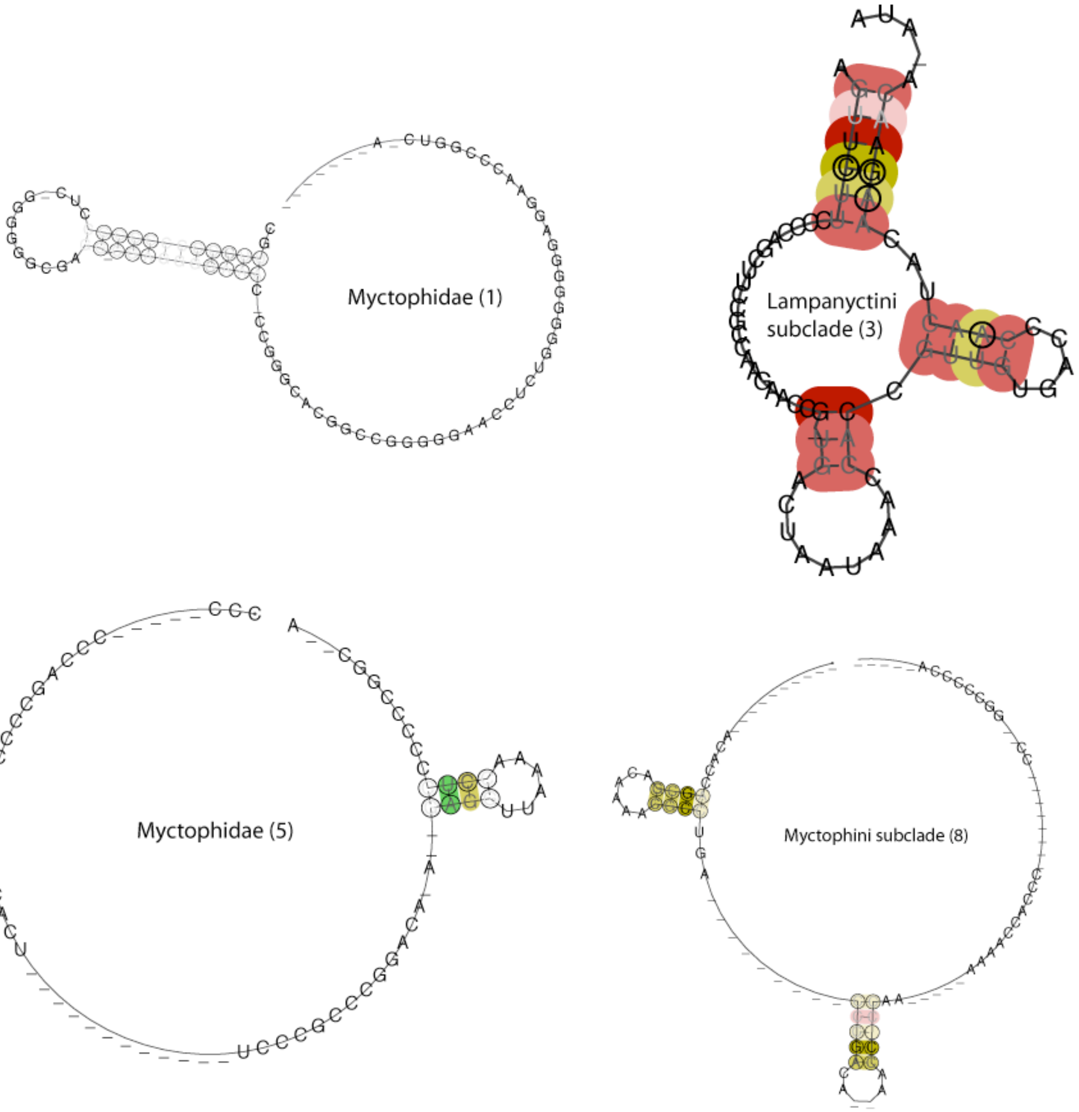

Supplement: Additional file 3: Figure S1 — Secondary structure of INC-regions. Sequence alignments and secondary tRNA structures of INC-regions are shown for synapomorphic spacers pertaining to gene order rearrangements 1, 3, 5 and 8 presented in Figure 3. The remaining INC-regions presented in Figure 3 are relatively short sequences and are not included. [file 1471-2148-13-111-S3.pdf]
