# Supplementary material for: Mitogenomic sequences and evidence from unique gene rearrangements corroborate evolutionary relationships of myctophiformes (Neoteleostei)
Source: BMC Evol Biol. 2013 Jun 3;13:111. doi: 10.1186/1471-2148-13-111 (PMC3682873; doi:10.1186/1471-2148-13-111)
Supplement: Additional file 4: Table T1 — Base pair details of INC-regions found in Myctophidae. Eight mt regions correspond to gene order and INC-region synapomorphies presented in Figure 3 with slash (/) denoting bordering genes. The last column includes mt information from the polymorphic region from tRNA-Thr and downstream towards the control region (CR). Numbers of INC-sequence base pairs are parenthesized next to flanking genes. Greater-than sign (>) and hyphen (−) denote partial and no sequence, respectively, from failure to complete the sequences. [file 1471-2148-13-111-S4.pdf]

Classification	Species	1. $WAN_{OL}CY$	2. IQM-region	3. ND6/Cytb	4. ND5/Cytb
Neoscopelidae	*Solivomer arenidens*				
	Scopelengys tristis				
	Neoscopelus macrolepidotus				
	Neoscopelus microchir				
Notolychnini	*Notolychnus valdiviae*	WAN(67)YC(27)	I(21)M(27)QM		
Diaphini	*Lobianchia gemellarii*	WAN(68)YC(19)	I(12)M(43)Q(68)		
	Diaphus theta	WAN(90)YC(18)	IM(26)Q(62)		
	Diaphus luetkeni	WAN(88)YC(17)	IMQ(64)		
	Diaphus splendidus	WAN(86)YC(17)	IM(12)Q(62)		
	Diaphus gigas	WAN(87)YC(17)	IM(13)Q(64)		
	Diaphus Chrysorhynchus	WAN(86)YC(16)	IMQ(64)		
Gymnoscopelini	*Gymnoscopelus nicholsi*	WAN(87)Y(32)C(20)			
	Notoscopelus japonicus	WAN(89)YC(18)			
	Notoscopelus kroyeri	WAN(321)Y(26)C(91)			
	Notoscopelus caudispinosus	WAN(86)Y(25)C			
Lampanyctini	*Ceratoscopelus maderensis*	WAN(87)YC(14)			
	Bolinichthys pyrsobolus	WAN(87)YC(24)			
	Bolinichthys distofax	WAN(87)YC(17)			
	Taaningichthys minimus	WAN(93)YC(15)			
	Lampadena urophaos atlantica	WA(32)N(80)YC(13)			
	Lampadena yaquinae	WAN(89)YC(13)			
	Lampadena anomala	WAN(89)YC(11)			
	Stenobrachius leucopsarus	WAN(90)YC(13)		73	
	Triphoturus nigrescens	WAN(95)Y(17)C(14)		68	
	Nannobrachium ritteri	WAN(88)Y(18)C(13)		71	
	Lampanyctus macdonaldi	WAN(86)Y(12)C(15)		71	
	Lampanyctus intricarius	WAN(86)Y(12)C(13)		71	
Electronini	*Krefftichthys anderssoni*	(46)AN(444)ANT(17)C(15)			
	Protomyctophum arcticum	(35)A(39)YC(21)			
	Electrona antarctica	WAN(77)Y(17)C(14)			
Myctophini	*Diogenichthys atlanticus*	WAN(-)YC	IQMM		
	Benthosema glaciale	(19)WAN(56)YC(17)QM(117)	I(134)		
	Benthosema pterotum	WAN(83)YC(10)			
	Benthosema fibulatum	WAN(65)YC(12)			
	Symbolophorus californiensis	WAN(56)YC(14)			
	Myctophum orientale	WAN(79)YC(13)			
	Myctophum asperum	WAN(86)YC(12)			
	Myctophum punctatum	(13)WAN(68)Y(10)C(15)	IQ(94)M		17
	Myctophum nitidulum	WAN(84)Y(9)C(14)			17
	Myctophum affine	WAN(79)Y(11)C(14)	(89)IQM		16
Gonichthyini	*Centrobranchus choerocephalus*	WAN(88)Y(11)C(14)			

Classification	Species	5. L_1/ND1	6. ATP6/CO3	7. ND1/I	8. CO3/G	T and downstream
Neoscopelidae	*Solivomer arenidens*					T(22)P/CR
	Scopelengys tristis					T(11)P/CR
	Neoscopelus macrolepidotus					T(17)P/CR
	Neoscopelus microchir					T(18)P/CR
Notolychnini	*Notolychnus valdiviae*	62	288			-
Diaphini	*Lobianchia gemellarii*	66	18			T(157)P/CR
	Diaphus theta	62	19			-
	Diaphus luetkeni	62	17			T(>615)
	Diaphus splendidus	61	19			-
	Diaphus gigas	60	17			T(>123)
	Diaphus Chrysorhynchus	60	19			T(>500)
Gymnoscopelini	*Gymnoscopelus nicholsi*	73	25			-
	Notoscopelus japonicus	75	25			T(38)P/CR
	Notoscopelus kroyeri	72	25			T(136)P/CR
	Notoscopelus caudispinosus	79	25			T(39)P/CR
Lampanyctini	*Ceratoscopelus maderensis*	59	19			-
	Bolinichthys pyrsobolus	67	24			-
	Bolinichthys distofax	67	24			-
	Taaningichthys minimus	46	28			-
	Lampadena urophaos atlantica	72	26			T(77)P/CR
	Lampadena yaquinae	73	25			T(81)P/CR
	Lampadena anomala	73	25			T(81)P/CR
	Stenobrachius leucopsarus	70	24			-
	Triphoturus nigrescens	40	24			-
	Nannobrachium ritteri	70	24			T(109)E(54)P/CR
	Lampanyctus macdonaldi	69	23			T(>29)
	Lampanyctus intricarius	68	23			T(105)E(>61)
Electronini	*Krefftichthys anderssoni*	55	19	(206)IQM(9)		-
	Protomyctophum arcticum	49	19	-		-
	Electrona antarctica	54	19	(475)IQM	G(468)G	T(111)P/CR
Myctophini	*Diogenichthys atlanticus*	49	17		G(99)	-
	Benthosema glaciale	(69)L_1(63)	17		(70)G	-
	Benthosema pterotum	48	12		(46)G	T(28)P/CR
	Benthosema fibulatum	57	9		(59)G	T(43)P/CR

	Symbolophorus californiensis	54	18			-
	Myctophum orientale	48	17			T(28)P/CR
	Myctophum asperum	45	19			T(>247)
	Myctophum punctatum	58	17			T(>117)
	Myctophum nitidulum	54	16			T(104)ND6/E(37)P/CR
	Myctophum affine	58	16			T(98)ND6/E(22)P/CR
Gonichthyini	*Centrobranchus choerocephalus*	50	43			T(>373)
