# Supplementary material for: Mitogenomic sequences and evidence from unique gene rearrangements corroborate evolutionary relationships of myctophiformes (Neoteleostei)
Source: BMC Evol Biol. 2013 Jun 3;13:111. doi: 10.1186/1471-2148-13-111 (PMC3682873; doi:10.1186/1471-2148-13-111)
Supplement: Additional file 5 — IMQMψ gene order in a clade of scarid parrotfishes. Taxa constituting a monophyletic clade in Scaridae all show similar patterns in the IMQ-region as observed within Diaphini. Grey areas show INC-regions with number of base pairs noted for each taxon and the arrow indicates possible gradual removal of INC-regions. [file 1471-2148-13-111-S5.pdf]

Scarid parrotfishes

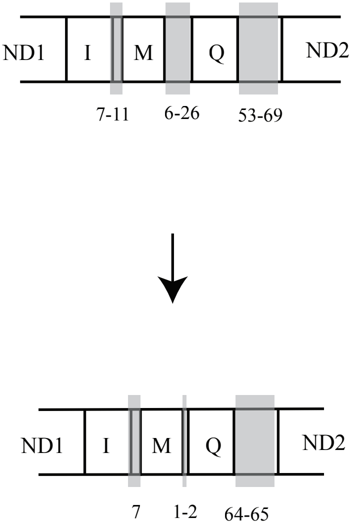

Scaridae

- *Calotomus japonicus 10 + 6 + 69*
- *Cetoscarus bicolor 11 + 6 + 64*
- *Chlorurus sordidus 7 + 9 + 66*
- *Leptoscarus vaigiensis 10 + 11 + 62*
- *Nicholsina usta 8 + 11 + 60*
- *Scarus ghobban 7 + 11 + 65*
- *Scarus guacamaia 7 + 15 + 65*
- *Scarus rubroviolaceus 7 + 11 + 65*
- *Scarus schlegeli 7 + 26 + 65*
- *Sparisoma chrysopterum 11 + 10 + 53*

Scaridae

- *Scarus forsteni 7 + 1 + 64*
- *Scarus niger 7 + 1 + 65*
- *Scarus oviceps 7 + 2 + 65*
